# Stress‐inducible phosphoprotein 1 (Sti1/Stip1/Hop) sequesters misfolded proteins during stress

**DOI:** 10.1111/febs.17389

**Published:** 2024-12-30

**Authors:** Benjamin S. Rutledge, Young J. Kim, Donovan W. McDonald, Juan C. Jurado‐Coronel, Marco A. M. Prado, Jill L. Johnson, Wing‐Yiu Choy, Martin L. Duennwald

**Affiliations:** ^1^ Department of Biochemistry The University of Western Ontario London Canada; ^2^ Department of Anatomy and Cell Biology The University of Western Ontario London Canada; ^3^ Robarts Research Institute and Department of Physiology and Pharmacology The University of Western Ontario London Canada; ^4^ Department of Biological Sciences University of Idaho Moscow ID USA

**Keywords:** co‐chaperone, protein homeostasis, scaffolding, Sti1, yeast

## Abstract

Co‐chaperones are key elements of cellular protein quality control. They cooperate with the major heat shock proteins Hsp70 and Hsp90 in folding proteins and preventing the toxic accumulation of misfolded proteins upon exposure to stress. Hsp90 interacts with the co‐chaperone stress‐inducible phosphoprotein 1 (Sti1/Stip1/Hop) and activator of Hsp90 ATPase protein 1 (Aha1) among many others. Sti1 and Aha1 control the ATPase activity of Hsp90, but Sti1 also facilitates the transfer of client proteins from Hsp70 to Hsp90, thus connecting these two major branches of protein quality control. We find that misbalanced expression of Sti1 and Aha1 in yeast and mammalian cells causes severe growth defects. Also, deletion of *STI1* causes an accumulation of soluble misfolded ubiquitinated proteins and a strong activation of the heat shock response. We discover that, during proteostatic stress, Sti1 forms cytoplasmic inclusions in yeast and mammalian cells that overlap with misfolded proteins. Our work indicates a key role of Sti1 in proteostasis independent of its Hsp90 ATPase regulatory functions by sequestering misfolded proteins during stress.

## Graphical abstract

Co‐chaperones are key elements in cellular protein quality control. They cooperate with the major heat shock proteins in folding and preventing the toxic accumulation of misfolded proteins upon exposure to stress. Stress‐inducible phosphoprotein 1 (Sti1/Stip1/Hop) is a major co‐chaperone to both Hsp90 and Hsp70. We find that misbalanced expression of Sti1 in yeast and mammalian cells causes severe growth defects. Deletion of *STI1* causes an accumulation of soluble misfolded ubiquitinated proteins and a strong activation of the heat shock response. During proteostatic stress, Sti1 associates with and sequesters soluble misfolded proteins in yeast and mammalian cells, scaffolding the proteostasis network.

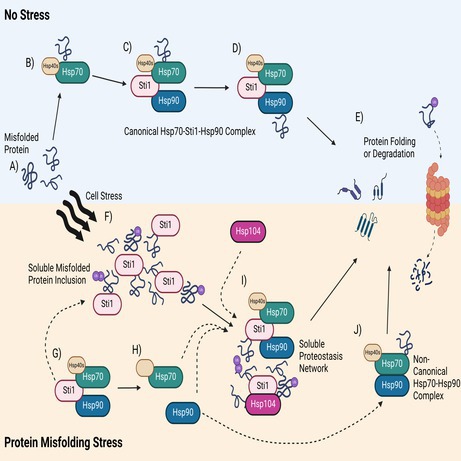

**Abbreviations**
AD: Alzheimer's disease
Aha1: activator of Hsp90 ATPase protein 1
ATP: adenosine triphosphate
ATP: adenosine triphosphate
AZC: L‐azetidine‐2‐carboxylic acid
BSA: bovine serum albumin
DMEM: Dulbecco's modified Eagle medium
DTT: dithiothreitol
Edc3: enhancer of mRNA‐decapping protein 3
EDTA: ethylenediaminetetraacetic acid
EDTA: ethylenediaminetetraacetic acid
EV: empty vector
FBS: fetal bovine serum
GAL: galactose
HCN: high copy number
Hop: Hsp‐organization protein
Hsf1: heat shock factor 1
Hsp: heat shock protein
HSRE: heat shock response element
LCN: low copy number
Luc: firefly luciferase protein
mRNA: messenger ribonucleic acid
Msn2/4: multicopy suppressor of SNF1 mutation proteins 2 and 4
Pab1: polyadenylate‐binding protein 1
PBS: phosphate‐buffered saline
PrP^C^
: cellular prion protein
SD: selective dextrose media
SDS: sodium dodecyl sulfate
SGal: selective galactose media
SGlyc: selective glycerol media
SKoAc: selective potassium acetate media
Sti1/Stip1: stress‐inducible phosphoprotein 1
STRE: stress response element
TBST: tris‐buffered saline with Tween‐20
TDP‐43: TAR DNA‐binding protein 43
TPR: tetratricopeptide repeat domain
YPD: yeast–peptone–dextrose

## Introduction

Molecular chaperones facilitate proper folding of nascent proteins, refold misfolded proteins under normal conditions or upon exposure to stress, and contribute to protein degradation [1, 2, 3]. The heat shock protein family (Hsps) includes the two most ubiquitously expressed molecular chaperones in eukaryotes, Hsp70 and Hsp90 [1, 4, 5]. Most Hsps, including Hsp90 and Hsp70, are classified as molecular chaperones due to their foldase functions, that is, their ATP hydrolysis‐driven folding of client proteins [6, 7]. Yet, some molecular chaperones, such as small heat shock proteins, can also function as holdases, that is, they sequester misfolded proteins and thus prevent toxicity [8].

Hsp90 undergoes a cycle of conformational changes upon binding and hydrolysis of ATP, which is crucial for its foldase function. Co‐chaperones, such as stress‐inducible phosphoprotein 1 (Sti1), the yeast homolog of Hsp70‐Hsp90 organizing protein (Hop) in humans, and activator of Hsp90 ATPase protein 1 (Aha1) regulate Hsp90 ATP hydrolysis, thereby regulating conformational changes of Hsp90 [9, 10, 11, 12]. Of note, Sti1 is one of the few co‐chaperones that interacts with both Hsp90 and Hsp70, which indicates a crucial function in connecting these two major chaperone systems [13]. Yet, Sti1 can also interact with other molecular chaperones independently of Hsp90, such as Hsp104 in yeast [14, 15]. The ability of Sti1 to interact with molecular chaperones independent of Hsp90 suggests that Sti1 may play a greater role in regulating chaperone interactions outside of its canonical Hsp90 co‐chaperone functions [16]. Approximately half of all reported physical interacting partners of Sti1 are involved in proteostasis [17], suggesting that Sti1 functions as a hub to coordinate different branches of cellular protein quality control, that is, protein folding and degradation. Sti1 seems also to be essential for the formation of the epichaperome complex, an integrated network comprised of molecular chaperones, clients, and co‐chaperones involved in proteostasis first identified in cancer cells [18].

Deletion of STI1 in *C. elegans* causes an impaired heat stress response [19], whereas in mice STIP1 knockout is embryonic lethal and even mouse embryonic fibroblasts are not viable [20]. Conversely, deletion of HOP in human HEK293T cancer cells does not reduce viability [21], although these cells are sensitized to cell stress due to reduced Hsf1 levels and activity [22]. Yeast cells deleted for STI1 grow normally under optimal growth conditions (30 °C, 2% glucose) [23], however also experience growth defects when exposed to protein misfolding stress (e.g., at 37 and 18 °C) [24]. Deletion of STI1 in yeast also directly reduces the availability of Hsp90 to its clients [25], causes hypersensitivity to Hsp90 inhibitors [26, 27], and prevents the formation of the Hsp90/Hsp70/Hsp104 and proteasome‐dependent heat‐induced inclusions [28]. Sti1 is also required for the K48‐ubiquitination‐mediated degradation of cytoplasmic proteins [29]. During periods of severe protein misfolding stress, Sti1 favors interactions with client proteins over its interactions with Hsp70 and Hsp90 [30].

Yeast Sti1 and human Hop are highly conserved, sharing similar domain structures and functions [31]. The interactions between Sti1/Hop and Hsp90 and Hsp70 are mediated by its three tetratricopeptide repeat domains (TPR1, TPR2a, and TPR2b). Both TPR1 and TPR2b contribute to binding Hsp70, with TPR1 being the primary binding site [31]. The TPR2a and TPR2b domains regulate Hsp90 ATPase inhibition [32, 33] via binding the middle domain of Hsp90. Even though Sti1 binds ATP and possesses slow ATPase activity on its own [34], its inability to refold clients in the absence of Hsp90 [35, 36] has prevented the classification of Sti1 as a molecular chaperone with foldase activity. As such, Sti1 is often regarded as an Hsp90 co‐chaperone without major independent functions. However, Sti1 has been demonstrated to play roles in diverse of cellular processes, including the regulation of endothelial cell polarization and migration, apoptosis, glycolysis, and regulation of the Wnt/β‐catenin pathway [37, 38, 39], without direct evidence of Hsp90 involvement. Human Aha1 has been classified as a holdase due to its interactions with client proteins via its amino terminal domain [40], whereas the yeast Aha1 does not possess such a client interacting domain and seems to operate solely to accelerate ATP hydrolysis by Hsp90 [41].

Here we compare the functions of Sti1 with yeast Aha1 under conditions of proteostatic stress. We demonstrate that yeast and mammalian cells require balanced levels of Sti1 and Aha1 for normal growth and particularly for survival under proteostatic stress. We also found that cells deleted for STI1 and AHA1 elicit a strong heat shock response, while the deletion of STI1 reduces the activity of the general stress response. Also, in the absence of Sti1, cells accumulate soluble ubiquitinated proteins. We further observed that in cells exposed to proteostatic stress, the subcellular localization of Sti1 changes from even distribution in the cytoplasm to distinct cytoplasmic foci and increased nuclear localization. Our results contribute to mounting evidence suggesting a larger role of Sti1 in sequestering misfolded proteins and promoting the formation of an integrated chaperone network to facilitate the clearance of soluble misfolded proteins during cellular stress conditions.

## Results

### Imbalance of Sti1 and Aha1 levels reduce yeast cell growth

**Fig. 1:**
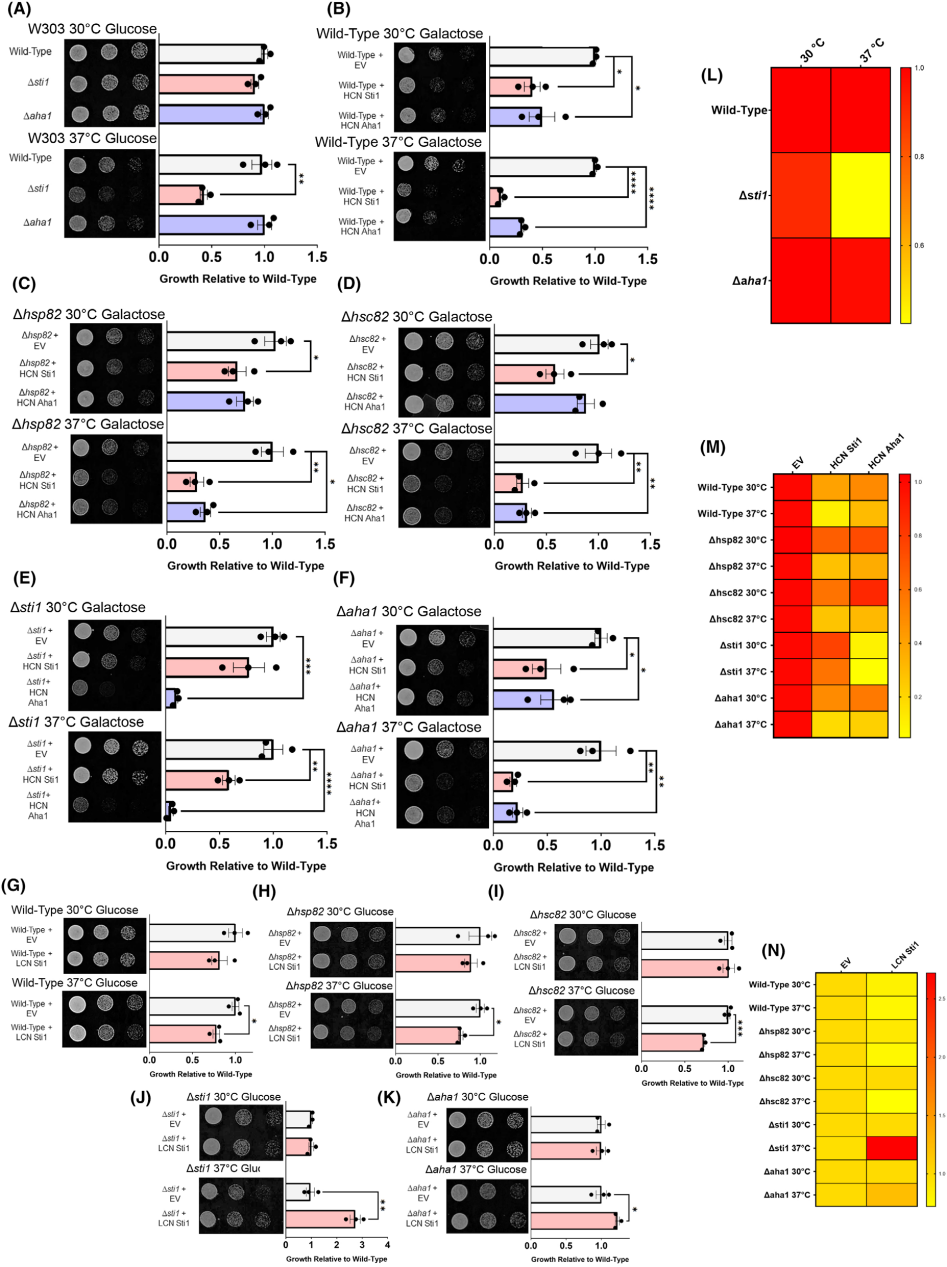
Imbalance of Sti1 and Aha1 induce growth defects in yeast. (A) Spotting assay of wild‐type, *∆sti1*, and *∆aha1* (W303) yeast grown at optimal growth temperatures and heat stress conditions (37 °C) on SD media. Cells are spotted in three fivefold dilutions from left to right and toxicity is inferred by lack of growth. (B–F) Spotting assays of (B) wild‐type, (C) *Δhsp82*, (D) *Δhsc82*, (E) *∆sti1*, and (F) *∆aha1* yeast (BY4741) transformed with high copy number (HCN) empty vector (EV) or expression plasmids of Sti1 or Aha1 and grown at optimal growth temperatures and heat stress conditions (37 °C) on selective galactose (SGal) media. (G–K) Spotting assays of (G) wild‐type, (H) *Δhsp82*, (I) *Δhsc82*, (J) *∆sti1*, and (K) *∆aha1* yeast (BY4741) transformed with low copy number (LCN) EV or LCN expression plasmids of Sti1 and grown at optimal growth temperatures (30 °C) and heat stress conditions (37 °C) on selective dextrose (SD) media. (L–N) Quantification of (L) *∆sti1* and *∆aha1* yeast growth and (M) HCN or (N) LCN overexpression. For all growth assays, three biological replicates were used. To determine statistical significance, unpaired *t*‐tests were used to compare means and standard deviations between relevant controls and experimental data sets (each data set was composed of a minimum of three biological replicas). Statistical significance is represented by an asterisk, where **** is *P* < 0.0001, *** is *P* < 0.001, ** is *P* < 0.01, and * is *P* < 0.05. Error bars represent standard errors of the mean.

To determine if Sti1 possesses functions beyond its role as a Hsp90 co‐chaperone, we performed genetic assessment of yeast strains ablated for or overexpressing Sti1. Since Aha1 in yeast lacks any known client interaction capacity, we used it as a control. We first assessed how the deletion and overexpression of Sti1 and Aha1 alter growth in yeast cells either exposed to proteostatic stress or in cells bearing deletions of HSP82, HSC82, AHA1, and STI1. In agreement with previous studies [24, 42], we observe a significant growth reduction in cells deleted for STI1 and no observable growth reduction for cells deleted for AHA1 under heat stress (growth at 37 °C) (Fig. 1A,L and Fig. S1). In addition, when yeast cells are chronologically aged, *∆sti1* cells show growth defects during early stages of aging, whereas *∆aha1* cells show growth defects only after prolonged periods of aging (Fig. S2).

To determine how excess Sti1 and Aha1 protein levels affect cell growth, we overexpressed both co‐chaperones using a strong, galactose inducible promoter and a high copy number (HCN) expression plasmid (2 micron). When grown on plates supplemented with 2% galactose as the sole carbon source (inducing conditions), cells expressing the inducible high copy number Sti1 expression plasmid exhibit and increase in Sti1 steady‐state expression levels by almost 30‐fold compared to control cells (Fig. S3). Notably, endogenous Sti1 steady‐state levels are significantly reduced when cells are grown in media containing galactose as compared to cells grown in glucose (Fig. S3), which may be explained by a link between the Hsp90 and Hsp70 and the expression of galactose inducible genes [43].

In wild‐type yeast cells, high copy number Sti1 and Aha1 expression significantly reduce growth at 30 °C and causes an even more severe growth reduction at 37 °C (Fig. 1B,L). To test if the overexpression of Sti1 and Aha1 cause reduced growth through their interactions with Hsp82 or Hsc82, high copy number expression plasmids of Sti1 and Aha1 were transformed into *Δhsp82* or *Δhsc82* yeast cells. At 30 °C high copy number Sti1 expression causes a significant growth reduction relative to vector controls in *Δhsp82* or *Δhsc82* yeast, respectively, whereas Aha1 overexpression in *Δhsp82* or *Δhsc82* yeast does not significantly reduce growth at 30 °C (Fig. 1C,D). However, both high copy number Sti1 and Aha1 expression cause severe growth defects in *Δhsp82* and *Δhsc82* when grown at 37 °C (Fig. 1C,D). These results suggest that unlike Aha1, the reduction in cell growth caused by high Sti1 overexpression is at least partially independent of expression of Hsp82 or Hsc82.

To further explore the effects of imbalanced levels of Hsp90 co‐chaperones, we transformed high copy number Sti1 and Aha1 expression plasmids into *∆sti1* and *∆aha1* yeast and assessed growth. Expression of Sti1 using the high copy number plasmid in *∆sti1* yeast does not significantly reduce growth at 30 °C, however, significantly reduces growth at 37 °C (Fig. 1E), indicating that expression levels exceed those of endogenous Sti1. In *∆aha1* yeast, high copy number Aha1 expression causes similar growth defects to that of high copy number Aha1 expression in the wild‐type strain (Fig. 1F). Furthermore, high copy number expression of Aha1 in *∆sti1* cells causes a severe growth reduction at both 30 and 37 °C (Fig. 1E). Conversely, high copy number Sti1 expression in *∆aha1* yeast causes a growth reduction at 30 and 37 °C, which is comparable to the growth reduction observed by high copy number Sti1 expression in wild‐type, *Δhsp82*, or *Δhsc82* strains (Fig. 1F). Taken together, these results show that the deletion of Sti1 enhances the growth defects caused by high copy number Aha1 expression, whereas deletion of AHA1, HSP82, and HSC82 does not affect the growth defects associated with overexpression of Sti1 (Fig. 1M).

The extremely high overexpression of our high copy number Sti1 expression plasmids may create a drastic imbalance within the proteostasis network of the cell, exacerbating the observed phenotypes. We thus sought to assess how moderate overexpression of Sti1 from a low copy number plasmid (centromeric, LCN with the weaker constitutive GPD promotor [44] driving Sti1 and Aha1 expression) affects growth. The low copy number expression system causes only a 2.1‐fold increase in Sti1 expression relative to endogenous Sti1 levels in SD media (Fig. S3). Low copy number Sti1 expression does not induce growth defects in all strains tested when grown at 30 °C (Fig. 1G–K,N).

To confirm that the lack of growth reduction in the low copy number system was not solely due to rapid cell division and a fermentation‐based metabolism, we conducted growth assays at 30 °C on nonfermentable carbon sources, such as glycerol and potassium acetate. Again, low copy number expression of Sti1 caused no significant growth reduction, indicating that the metabolic shift to oxidative phosphorylation does not affect the ability of cells to tolerate low copy number Sti1 expression in the absence of stress (Fig. S4). By contrast, when cells are grown at 37 °C in SD, fermentable, media, low copy number Sti1 expression causes a comparable growth reduction in wild‐type, *Δhsp82*, or *Δhsc82* yeast (Fig. 1G–I,N). This indicates that moderate overexpression of Sti1 reduces cell growth during stress independent of Hsp82 and Hsc82 levels as we observed for the high copy number expression system. Low copy number expression of Sti1 in the *∆sti1* strain rescued growth defects during heat stress (Fig. 1J,N). Unexpectedly, however, low copy number Sti1 expression in the *∆aha1* strain also rescued growth under heat stress (Fig. 1K,N). No growth defect was observed in the *∆aha1* strain compared to wild‐type yeast during heat stress.

Taken together, these results indicate that imbalanced levels of Sti1 and Aha1 reduce cell growth (Fig. 1B). Deletion of Hsp82 or Hsc82 reduces the growth phenotype of high Aha1 overexpression at 30 °C (Fig. 1C,D) but does not protect against high or moderate Sti1 overexpression at 30 or 37 °C (Fig. 1C,D,H,I), suggesting that Sti1 can reduce cell growth independent of its interaction with Hsp90.

### The C‐terminal domains TPR2a, TPR2b, and DP2 of Sti1 are key for its toxicity

**Fig. 2:**
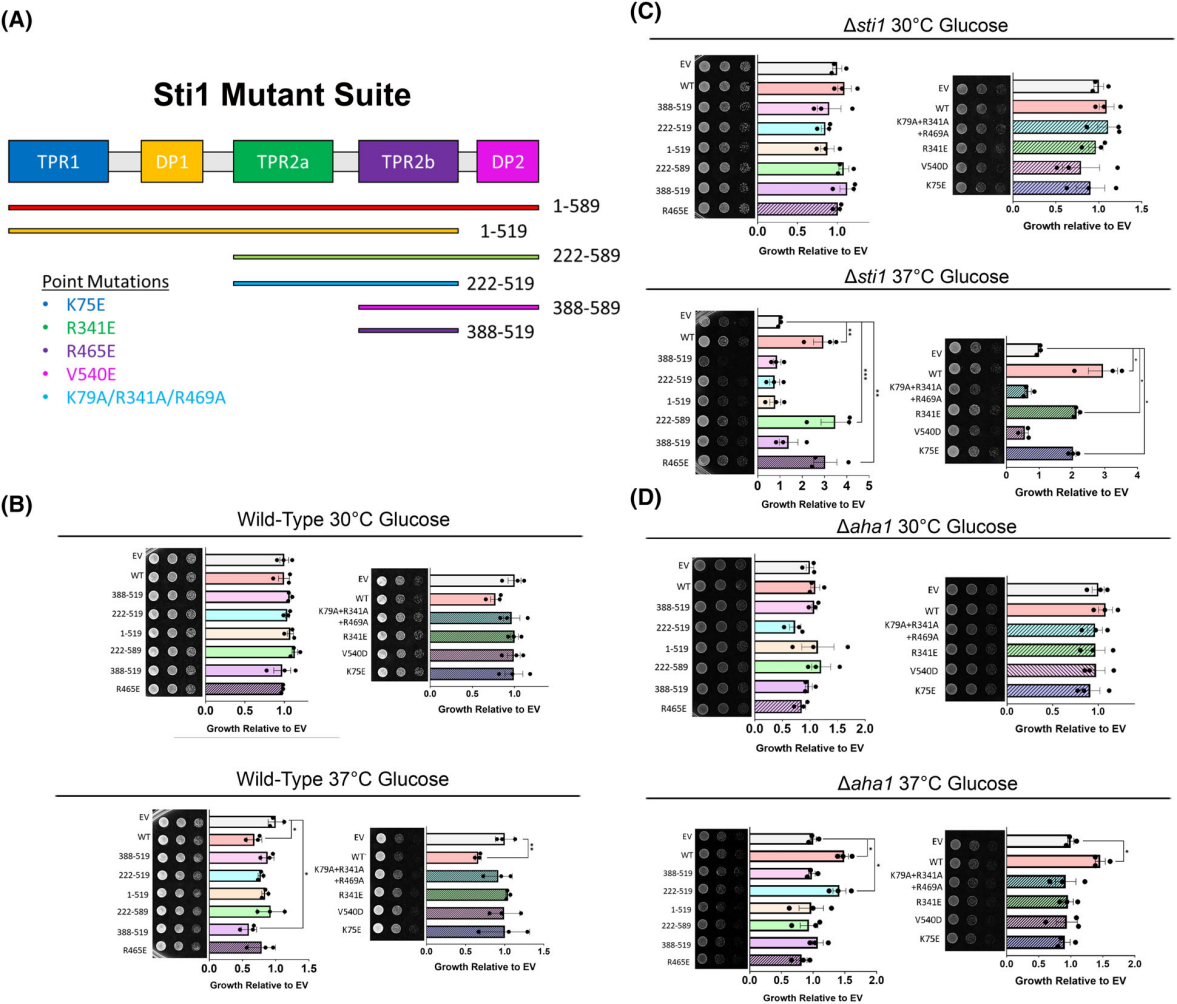
Domain characterization of Sti1 overexpression‐associated growth defects. (A) Domain map of Sti1 and depiction of domain constructs and function disrupting point mutations. (B–D) Yeast growth assay of (B) wild‐type, (C) *∆sti1*, and (D) *∆aha1* yeast (W303) transformed with low copy number (LCN) empty vector (EV) or expression plasmids of Sti1 constructs and grown at optimal growth temperatures and heat stress conditions (37 °C). Cells are spotted in three fivefold dilutions from left to right and toxicity is inferred by lack of growth. For all growth assays, three biological replicates were used. To determine statistical significance, unpaired *t*‐tests were used to compare means and standard deviations between relevant controls and experimental data sets (each data set was composed of a minimum of three biological replicas). Statistical significance is represented by an asterisk, where **** is *P* < 0.0001, *** is *P* < 0.001, ** is *P* < 0.01, and * is *P* < 0.05. Error bars represent standard errors of the mean.

To determine which specific domains of Sti1 are responsible for the toxicity associated with Sti1 overexpression, we tested a suite of previously established low copy number expression plasmids encoding Sti1 truncations lacking different domains, Sti1 mutants altering key basic residues in the Hsp70/Hsp90‐binding cleft of the TPR1, TPR2a, and TPR2b domains (K75E, R341E, and R465E, respectively), a combination of TPR mutations (K79A + R341A + R469A), and a mutation of a conserved residue in the DP2 domain (V540E) [31] (Fig. 2A). In wild‐type cells, only overexpression of the TPR2b domain (amino acid position 388–519) caused growth defects similar to the overexpression of full‐length Sti1 in cells grown at 37 °C (Fig. 2B). In accordance with previously published results [31], we observed that in addition to full‐length wild‐type Sti1, Sti1 containing only the TPR2a, TPR2b, and DP2 domains (amino acid position 222–589) is sufficient to complement the deletion of STI1 during heat stress. Furthermore, Sti1 containing mutations in either the TPR2a or TPR2b, but not in both domains together, can complement the growth defect observed in *∆sti1* cells (Fig. 2C) indicating functional redundancy between TPR2a and TPR2b. In the *∆aha1* strain, only expression of full‐length wild‐type Sti1 and the TPR2a and TPR2b domains could produce the growth defect we observed at 37 °C (Fig. 2D). Taken together, these results further support previous claims that the interaction between Hsp70 and TPR1 is functionally dispensable due to the higher affinity binding between Hsp70 and TPR2B [45]. Moreover, both the TPR1 and DP1 domains are largely expendable for Sti1 function [31], as neither domain is required to rescue the growth defect of *∆sti1* yeast grown at 37 °C. However, our results show that the TPR1 and DP1 domains may be involved in mediating the growth defects associated with Sti1 overexpression.

### Deletion of STI1 or AHA1 induce the heat shock response

**Fig. 3:**
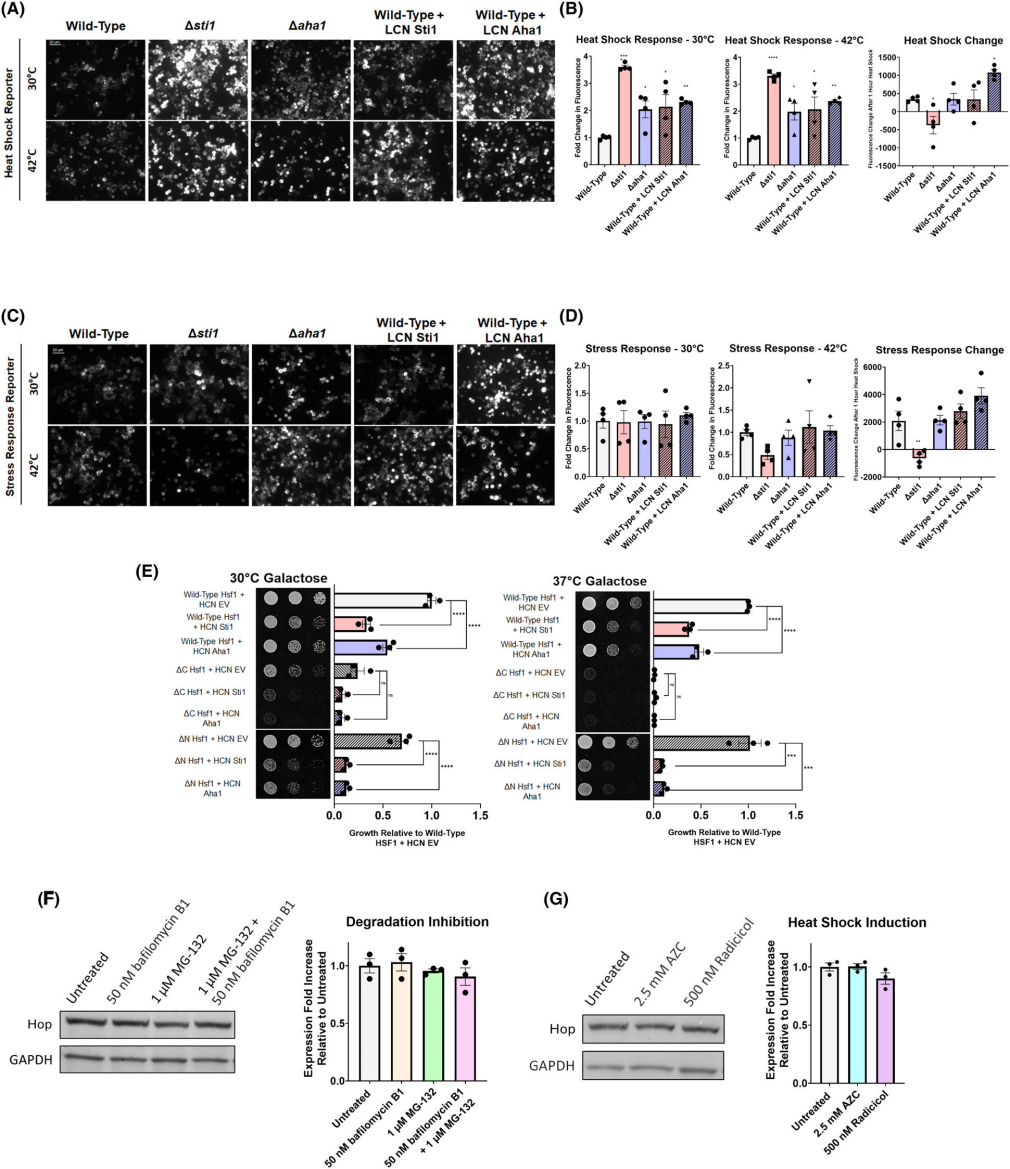
Deletion of STI1 elicits the heat shock response. (A–D) Fluorescence microscopy and reporter assay quantification of wild‐type, *∆sti1*, and *∆aha1* yeast transformed with low copy number (LCN) expression plasmids of Sti1 or Aha1 and fluorescent reporter plasmids of (A, B) heat shock element induction and (C, D) stress response element induction before and after 1 h of heat shock (42 °C) in selective dextrose (SD) media. For all fluorescent reporter assays, four biological replicates were used. Scale bars represent 10 μm. (E) Growth assays of BY 4741 yeast cells expressing either wild‐type heat shock factor 1 (HSF1), nonfunctional HSF1 (ΔC), or constitutively active HSF1 (ΔN) in place of the endogenous HSF1 gene transformed with high copy number (HCN) expression plasmids of Sti1 or Aha1 and grown at optimal growth temperatures and heat stress conditions (37 °C) on selective galactose (SGal) media. Cells are spotted in three fivefold dilutions from left to right and toxicity is inferred by lack of growth. For all growth assays, three biological replicates were used. (F, G) Quantification of Sti1 protein levels determined by western blot analysis using an anti‐Hop primary antibody in SH‐SY5Y cell lysates from cells grown under normal growth conditions or treated with reagents to (F) inhibit protein degradation or (G) induce heat shock. Western blots were also probed using an anti‐GAPDH primary antibody as a loading control. Quantification of Sti1 protein levels utilizes cell lysates from three biological replicates. To determine statistical significance, unpaired t‐tests were used to compare means and standard deviations between relevant controls and experimental data sets (each data set was composed of a minimum of three biological replicas). Statistical significance is represented by an asterisk, where *** is *P* < 0.001, ** is *P* < 0.01, and * is *P* < 0.05. Error bars represent standard errors of the mean.

Sti1 was first identified as a stress‐inducible protein on the basis that Sti1 overexpression induced expression of the heat shock‐controlled promoter of SSA4, one of the cytoplasmic Hsp70s in yeast [23]. We thus aimed to decipher how Sti1 and Aha1 alter the induction of the heat shock response and the general stress response pathways. To this end, we measured the expression of fluorescent protein reporters under control of heat shock response elements (HSREs) and stress response elements (STREs) [46]. In agreement with the Hsf1 activity screen performed in a genome‐wide loss‐of‐function yeast library by Brandman *et al*. [46], the HSRE reporter produces significantly more fluorescence in *∆sti1* cells, and to a lesser extent in *∆aha1* cells, compared to wild‐type cells when grown at 30 °C (Fig. 3A,B). By contrast, both *∆sti1* and *∆aha1* cells show a similar STRE reporter fluorescence compared to wild‐type cells when grown at 30 °C (Fig. 3C,D). After heat shock (42 °C for 1 h), *∆aha1* cells show a similar increase in HSRE reporter fluorescence to wild‐type cells, whereas fluorescence decreased in *∆sti1* cells after heat shock (Fig. 3A,B). Similarly, *∆aha1* cells show similar STRE reporter fluorescence after heat shock as compared to wild‐type cells, whereas *∆sti1* cells show reduced STRE reporter levels after heat shock as compared to wild‐type cells (Fig. 3C,D). We thus observe that deletion of Sti1 increases activation of the heat shock response to its maximum threshold under normal growth conditions. Also, we observe that deletion of Sti1 impairs the activation of the general stress response pathways mediated by Msn2/4 activity.

To determine how overexpression of Sti1 and Aha1 affects the activity of Hsf1 and Msn2/4, yeast cells were transformed with low copy number Sti1 or Aha1 plasmids and the HSRE and STRE reporter induction was measured. Low copy number expression of Sti1 and Aha1 both significantly increase HSRE reporter fluorescence at 30 °C. Low overexpression of Sti1 did not significantly increase the HSRE reporter fluorescence after heat shock compared to normal growth temperature (30 °C), whereas the low overexpression of Aha1 caused a significant increase in fluorescence compared to wild‐type cells after heat shock (42 °C for 1 h). Together, our results show that the overexpression of Sti1 and Aha1 cause increased activity of Hsf1 during normal growth, yet only overexpression of Aha1 increases Hsf1 activity after heat shock (Fig. 3A,B). By contrast, overexpression of Sti1 or Aha1 did not affect the STRE reporter fluorescence before or after heat shock, indicating that excess Sti1 or Aha1 does not affect Msn2/4 activity (Fig. 3C,D).

We further tested if increased activity of Hsf1 can compensate for the reduced growth of *∆sti1* cells during stress. To this end, we overexpressed Sti1 or Aha1 in yeast cells expressing either wild‐type Hsf1, Hsf1 missing its C‐terminal transactivation domain (ΔC), resulting in reduced activity, or Hsf1 missing the regulatory N‐terminal residues (ΔN), which is constitutively active [47]. When grown at 30 and 37 °C, high copy number expression of Sti1 or Aha1 in both the wild‐type Hsf1 and ΔN Hsf1 cells causes significant growth defects. High copy number expression of Sti1 and Aha1 in ΔC Hsf1 cells did not produce a significant change in growth compared to wild‐type cells. It is, however, important to note that growth in the ΔC Hsf1 background is already significantly reduced. Under heat stress the ΔN Hsf1 background shows similar growth to wild‐type, however, high copy number Sti1 or Aha1 expression causes a more severe growth defect in the ΔN Hsf1 background compared to the wild‐type background (Fig. 3E). These results indicate that manipulation of Hsf1 activation cannot compensate for the toxicity produced by the overexpression of Sti1 or Aha, and constitutive activation of Hsf1 even exacerbates the toxicity caused by overexpression of Sti1 and Aha1 during heat stress.

Since manipulating Sti1 expression increased Hsf1 activity, we investigated if Hop protein levels are regulated by Hsf1 activity in mammalian cells. The promotor regulating Hop contains heat shock elements (HSEs) and Hop expression was thus proposed to be under the transcriptional control of Hsf1 [48]. Hop mRNA levels are regulated in a similar manner to Hsp70 and Hsp90 in human cancer cells [49], yet the mechanisms underlying the regulation of Hop protein levels are not fully understood. We thus tested if stressors known to induce activity of genes under Hsf1 regulation increase Hop protein levels. Human neuroblastoma (SH‐SY5Y) cells were cultured in the proteasome inhibitor MG‐132 [50], bafilomycin B1 [51], radicicol [52], and AZC [53], all of which induce a robust heat shock response and Hop levels were quantified by western blot. Unlike other commonly used human cancer cell lines which often constitutively upregulate chaperone expression, chaperone levels are not constitutively upregulated in SH‐SY5Y cells and protein levels of chaperones, such as Hsp70, have been demonstrated to increase in response to stress [54]. None of the treatments tested produced significant changes in Hop protein levels (Fig. 3F,G), whereas we detected a significant increase in Hsp70 protein levels (Fig. S5). Our results indicate that Hop protein levels do not increase upon exposure to proteostatic stress.

### Deletion of STI1 causes the accumulation of soluble ubiquitinated proteins

**Fig. 4:**
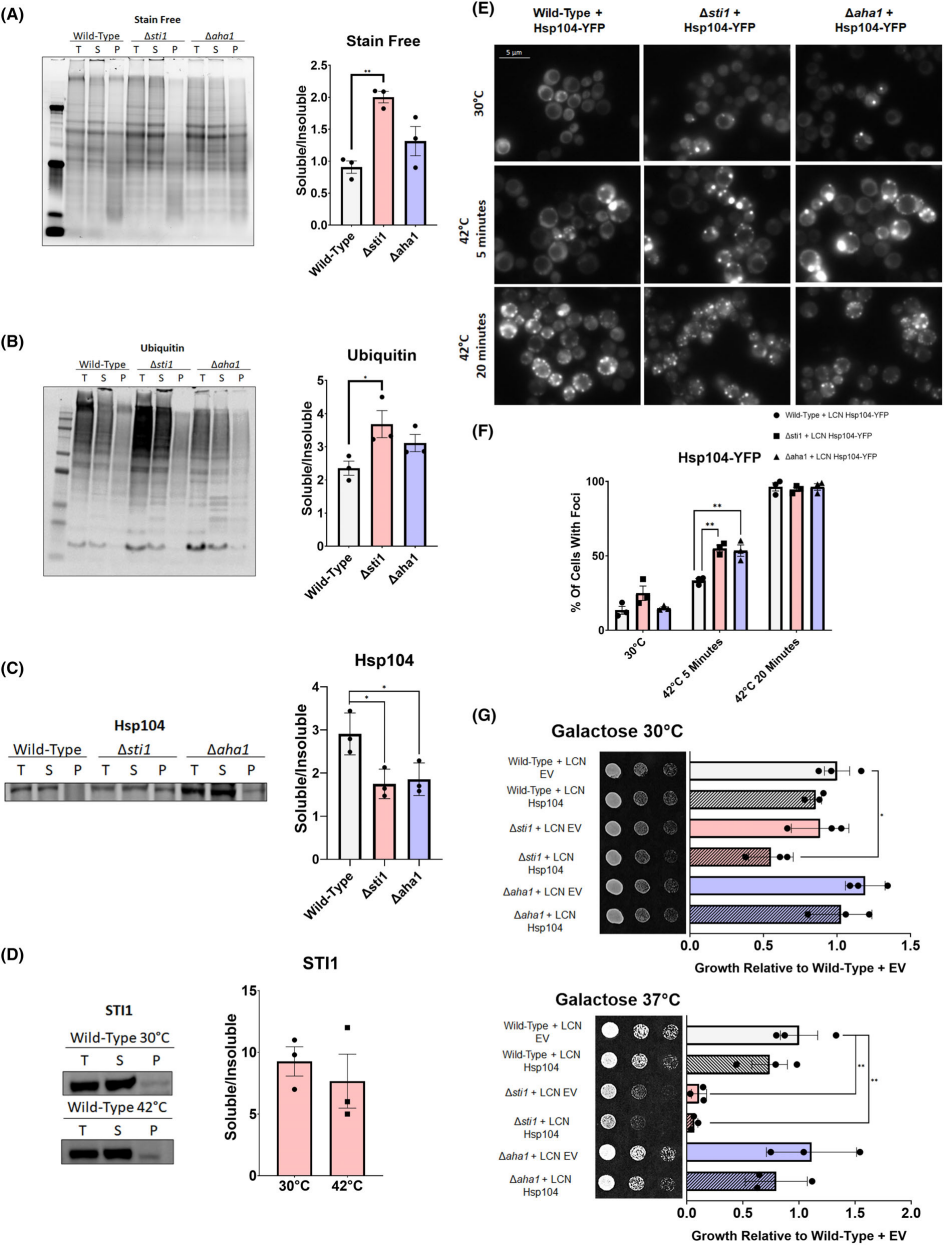
Deletion of STI1 causes an accumulation of ubiquitinated proteins. (A–C) Sedimentation analysis of protein lysates from wild‐type, *∆sti1*, and *∆aha1* yeast (W303) cells grown at 30 °C in yeast–peptone–dextrose (YPD) media. Fractionated lysates were imaged with (A) stain‐free gels and probed with (B) anti‐ubiquitin, and (C) anti‐Hsp104 primary antibodies for quantification of sedimentation profile. (D) Sedimentation analysis of protein lysates from wild‐type yeast (W303) cells grown at 30 °C or heat shocked for 1 h at 42 °C in YPD. Fractionated lysates were probed with anti‐Sti1 primary antibodies for quantification of sedimentation profile. Quantification of all sedimentation profiles utilize cell lysates from three biological replicates. (E) Fluorescence microscopy of wild‐type, *∆sti1*, and *∆aha1* yeast transformed with low copy number (LCN) plasmids expressing Hsp104‐YFP after various lengths of heat shock in selective dextrose (SD) media. Scale bars represent 5 μm. (F) Quantification of Hsp104 foci formation from three biological replicates. (G) Spotting assays of wild‐type, *∆sti1*, and *∆aha1* W303 yeast transformed with LCN expression plasmids of Hsp104 and grown at optimal growth temperatures and heat stress conditions (37 °C) on selective galactose (SGal) media. Cells are spotted in three fivefold dilutions from left to right and toxicity is inferred by lack of growth. For all growth assays, three biological replicates were used. To determine statistical significance, unpaired t‐tests were used to compare means and standard deviations between relevant controls and experimental data sets (each data set was composed of a minimum of three biological replicas). Statistical significance is represented by an asterisk, where **** is *P* < 0.0001, *** is *P* < 0.001, ** is *P* < 0.01, and * is *P* < 0.05. Error bars represent standard errors of the mean.

Next, we investigated if the ablation of Sti1 affects the accumulation of misfolded proteins in yeast. Our sedimentation assays show that STI1 deletion caused an increase in the ratio of soluble to insoluble proteins compared to wild‐type, whereas the deletion of AHA1 had no effect (Fig. 4A). Furthermore, the deletion of STI1 caused a significant increase in the ratio of soluble to insoluble ubiquitinated proteins, while the deletion of AHA1 again had no effect (Fig. 4B). Since Hsp104 interacts with Sti1 independently of Hsp90 [14], lysates were probed with anti‐Hsp104 antibodies to determine if the deletion of Sti1 alters how Hsp104 associates in the soluble or insoluble protein fraction. Both the deletion of STI1 and AHA1 caused a significant increase in the amount of Hsp104 in the insoluble fraction, confirming that Hsp104 favors interactions with insoluble client proteins, which is exacerbated in both deletion strains (Fig. 4C). When cells were heat shocked for 1 h at 42 °C before lysis, we detected no significant difference in the sedimentation distribution of total protein, ubiquitinated protein, or of Hsp104 between the deletion strains and wild‐type cells (Fig. S6A–C). Finally, we asked if the sedimentation profile of Sti1 changes in response to heat shock. Fractionated lysates from yeast grown at 30 °C or heat shocked for an hour at 42 °C were probed using an anti‐Sti1 primary antibody. The majority of Sti1 is found in the soluble fraction, and no significant shift to the insoluble fraction is observed after heat shock (Fig. 4D). Together these results suggest that STI1 modulates the accumulation of soluble ubiquitinated proteins, whereas deletion of STI1 shifts the equilibrium of the Hsp104 interactome to favor insoluble clients (Fig. 4A–D).

To test if the Hsp104 in the insoluble protein fraction interacts with insoluble client proteins in the STI1 or AHA1 deletion strains, plasmids expressing a Hsp104‐YFP fusion protein were transformed into wild‐type, *∆sti1*, and *∆aha1* yeast strains. Cells were heat‐shocked and fluorescence microscopy was performed to observe the number of cells containing Hsp104 foci. Before heat shock, the percent of *∆sti1* cells containing Hsp104‐YFP foci was not different from wild‐type cells. However, an increase in Hsp104‐YFP foci were observed in both *∆sti1* and *∆aha1* compared to wild‐type cells after a brief heat shock (Fig. 4E,F). The increase in Hsp104‐YFP foci in *∆sti1* cells also indicates that Sti1 is not required for the formation of these Hsp104 foci.

Since increased insoluble Hsp104 and Hsp104‐YFP foci are observed in the *∆sti1* strain, a galactose inducible low copy number Hsp104 plasmid was transformed into wild‐type, *∆sti1*, and *∆aha1* yeast strains to determine if increased Hsp104 levels suppress the growth phenotype of the *∆sti1* strain. At 30 °C, the overexpression of Hsp104 significantly reduced the growth of the *∆sti1* strain. At 37 °C, however, Hsp104 overexpression did not suppress the growth defect observed in the *∆sti1* strain, but rather increased it (Fig. 4G). Conversely, overexpression of Hsp104 in *∆aha1* yeast had no effect on growth at either 30 or 37 °C. Together, these results show that Hsp104 associates with insoluble clients in *∆sti1* yeast compared to wild‐type, and that Hsp104 overexpression exacerbates the growth defect of ∆*sti1* yeast rather than reducing it.

### Sti1 accumulates in the nucleus and forms cytoplasmic foci during cellular stress

**Fig. 5:**
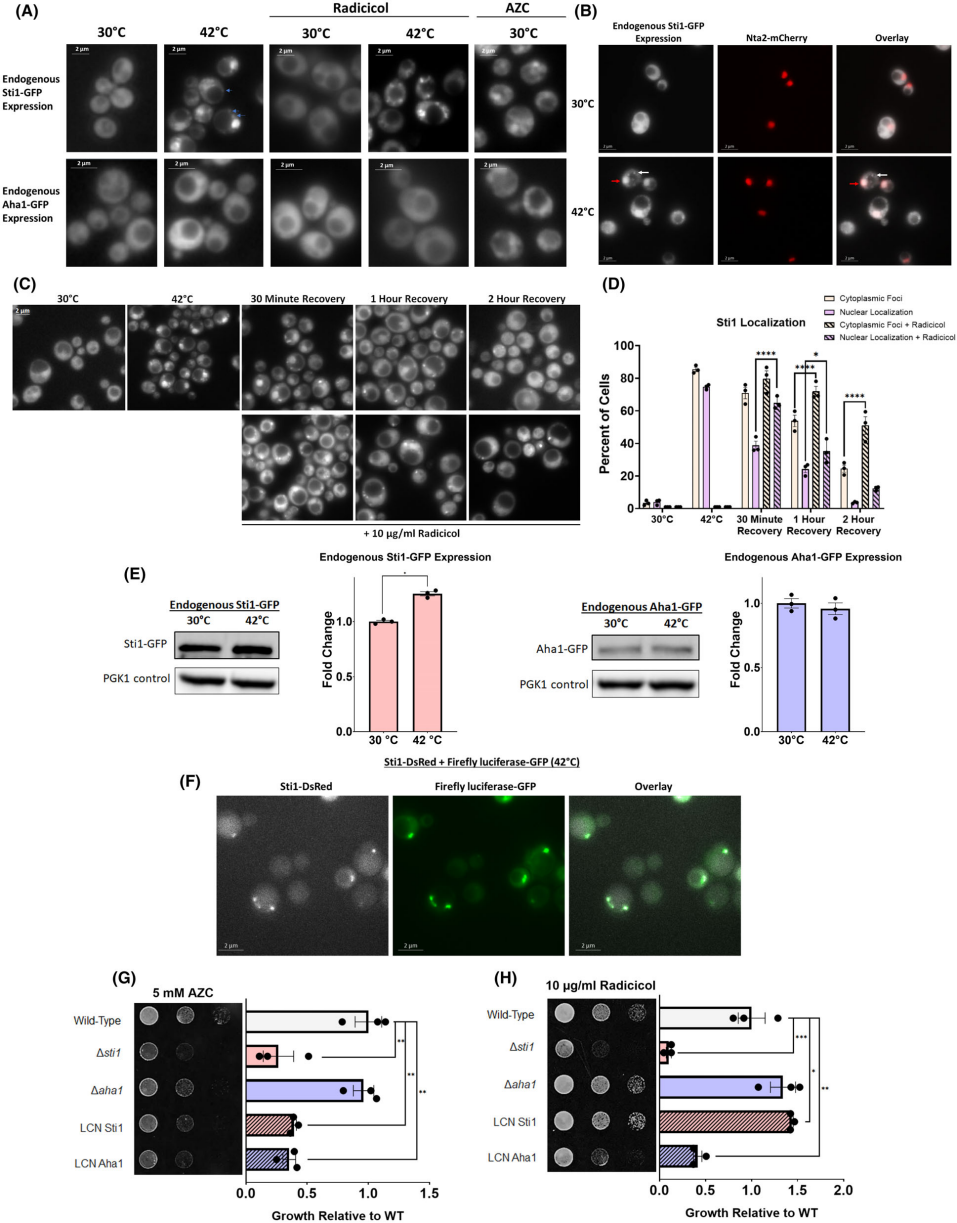
Sti1 translocates to the nucleus and forms cytoplasmic inclusions during protein misfolding stress. (A) Fluorescence microscopy of yeast (BY4741) cells expressing Sti1‐GFP or Aha1‐GFP under their endogenous promoters before and after a 1‐h heat shock (42 °C), and in the presence of radicicol and L‐azetidine‐2‐carboxylic acid (AZC) in selective dextrose (SD) media. Examples of cytoplasmic inclusions are highlighted with blue arrows. (B) Fluorescence microscopy before and after a 1‐h heat shock (42 °C) of yeast (BY4741) expressing Sti1‐GFP under its endogenous promoters and the nuclear protein NTA2‐mCherry on a transformed plasmid. Examples of nuclear localization and cytoplasmic foci of Sti1 are highlighted with red and white arrows, respectively. Yeast cells were grown in SD media overnight prior to heat shock. (C) Fluorescence microscopy of yeast (BY4741) expressing Sti1‐GFP under its endogenous promoter during recovery from a 1‐h heat shock (42 °C). Yeast cells were heat shocked and then incubated in SD media at 30 °C in the presence and absence of radicicol to monitor foci attenuation. Scale bars represent 2 μm. (D) Quantification of cytoplasmic foci and nuclear localization attenuation during recovery in the absence and presence of radicicol from three biological replicates. (E) Quantification of Sti1‐GFP and Aha‐GFP protein levels by western blot using an anti‐GFP primary antibody in cell lysates of yeast (BY4741) expressing Sti1‐GFP or Aha1‐GFP under their endogenous promoters before and after 1 h of heat shock (42 °C) in SD media. Quantification of Sti1 protein levels utilizes cell lysates from three biological replicates. (F) Fluorescence microscopy after 1 h of heat shock (42 °C) of yeast (W303) transformed with a low copy number (LCN) Sti1‐DsRed plasmid and a high copy number (HCN) Luc‐GFP plasmid. Scale bars represent 2 μm. (G, H) Growth assay of wild‐type, *∆sti1*, and *∆aha1* yeast (W303), and wild‐type yeast (W303) transformed with low copy number expression plasmids of Sti1 and Aha1, on SD media treated with (G) 5 mm AZC or (H) 10 μg·mL^−1^ radicicol at optimal growth temperatures (30 °C). For all growth assays, three biological replicates were used. To determine statistical significance, unpaired *t*‐tests were used to compare means and standard deviations between relevant controls and experimental data sets (each data set was composed of a minimum of three biological replicas). Statistical significance is represented by an asterisk, where **** is *P* < 0.0001, *** is *P* < 0.001, ** is *P* < 0.01, and * is *P* < 0.05. Error bars represent standard errors of the mean.

To further explore the role of Sti1 during proteostatic stress, we employed fluorescence microscopy of yeast strains expressing Sti1‐GFP or Aha1‐GFP fusion proteins integrated into the yeast genome at their endogenous loci [55]. When cells were grown at 30 °C in SD media, both Sti1‐GFP and Aha1‐GFP localized diffusely throughout the cytoplasm of the cells (Fig. 5A). Upon heat shock at 42 °C for an hour, Aha1‐GFP remains diffuse within the cytoplasm. By contrast, Sti1‐GFP forms distinct cytoplasmic foci in 85.4 ± 1.9% of cells and localizes to the nucleus in 74.7 ± 1.2% (Fig. 5A–D). To differentiate cytoplasmic foci from the stress‐induced nuclear translocation of Sti1 previously documented [56, 57], nuclear localization was confirmed by assessing the overlap of Sti1‐GFP with the nuclear marker Nta2‐mCherry (Fig. 5B). A time course analysis of cells during recovery from heat shock shows that after 2 h of recovery, Sti1 is no longer accumulated in the nucleus and only 24.5 ± 3.1% of cells still contain cytoplasmic foci (Fig. 5C,D). This indicates that both the nuclear accumulation and cytoplasmic inclusions of Sti1 rapidly appear upon heat stress but also rapidly reverse upon recovery from stress.

**Fig. 6:**
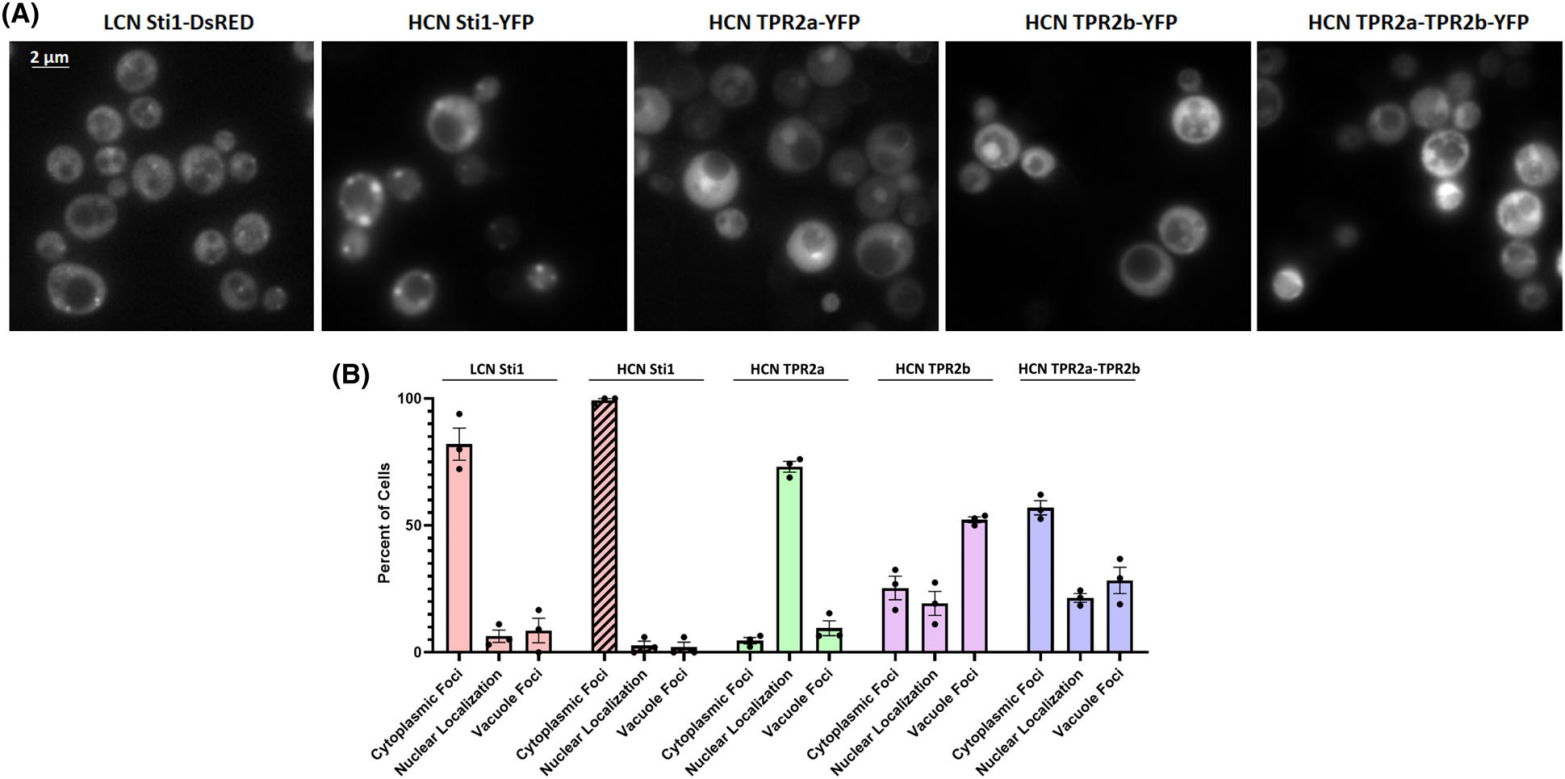
The TPR domains of Sti1 contribute to foci formation. (A) Fluorescence microscopy of yeast (W303) transformed with low copy number (LCN) Sti1‐DsRed and grown in selective dextrose (SD) media or transformed with high copy number (HCN) Sti1‐YFP, HCN tetratricopeptide repeat domains (TPR) TPR2a‐YFP (222–388), HCN TPR2b‐YFP (388–519), or TPR2a‐TPR2b‐YFP (222–519) and grown in selective galactose (SGal) media. Scale bars represent 2 μm. (B) Quantification of localization of Sti1 TPR domain constructs during overexpression in three independent biological replicates.

We next asked if the formation of cytoplasmic Sti1 foci at 42 °C is due to increased Sti1 protein levels resulting from induction of the heat shock response in yeast. Cells were heat shocked at 42 °C for 1 h and Sti1 protein levels were determined by western blot. During heat shock, Sti1 protein levels only minimally increase (1.25‐fold, Fig. 5E), which is similar to our results obtained in mammalian cells. These results also suggest that the formation of Sti1 foci cannot be caused merely by increased levels. We also tested if increased Sti1 expression in the absence of stress induces the formation of cytoplasmic Sti1 foci. Fluorescent microscopy reveals that yeast cells expressing both low copy number Sti1‐DsRed and high copy number Sti1‐YFP form cytoplasmic foci in the absence of stress (30 °C), suggesting overexpression of Sti1 can induce the formation of cytoplasmic foci in yeast. However, overexpression of Sti1 did not induce nuclear localization, supporting previous observations that nuclear localization of Sti1 during stress is regulated by factors such as post‐translational modifications [57], rather than protein levels (Fig. 6A,B).

Exposure of the proline analog AZC induces protein misfolding and the heat shock response and reduces the growth of cells with an impaired Hsp70 chaperone machinery [58, 59]. We thus sought to determine if manipulating Sti1 and Aha1 expression affects cellular growth under proteotoxic stress caused by AZC. The deletion of STI1 significantly reduced cell growth in the presence of AZC, whereas deletion of AHA1 had no effect. Surprisingly, low copy number overexpression of Aha1 or Sti1 also significantly reduced growth in the presence of AZC (Fig. 5G). Fluorescence microscopy of yeast cells expressing Sti1‐GFP or Aha1‐GFP under their endogenous promoters grown at 30 °C in the presence of AZC shows that both Sti1‐GFP and Aha1‐GFP fusion proteins form inclusions within the cytoplasm of the cells (Fig. 5A).

Hop can localize to stress granules in HeLa cells treated with hippuristanol, a small molecule that induces stress granule formation [60]. To test if the Sti1 foci, we observed during heat shock in yeast colocalize with stress granules or P bodies, Sti1‐DsRed or GFP fusions were expressed in yeast cells concomitant with stress granule marker Pab1‐GFP [61] or the P body marker Edc3‐DsRed [62]. Sti1 foci formed after heat shock (42 °C for an hour) do not overlap to great extent with either Pab1 or Edc3 foci, indicating that Sti1 does not strictly localize to stress granules or P bodies in yeast (Fig. S7B,C). We next investigated if Sti1 foci are associated with misfolded insoluble protein inclusions. We employed Hsp104‐RFP as a well‐established marker for accumulated insoluble misfolded proteins [63]. Yeast cells expressing Sti1‐GFP under the endogenous promoter were transformed with a low copy number plasmid expressing an Hsp104‐RFP. After heat shock at 42 °C for an hour, only partial overlap of Sti1 foci with Hsp104 was observed (Fig. S7A). Finally, to determine if Sti1 foci are associated with soluble inclusions of misfolded proteins, yeast cells were transformed with a Sti1‐DsRed plasmid and a plasmid encoding a GFP fusion of the thermolabile firefly luciferase protein (Luc‐GFP) that forms soluble cytoplasmic inclusions during heat stress [64, 65]. Upon heat shock, we observe extensive overlap of Sti1‐DsRed and Luc‐GFP foci, suggesting that Sti1 is associated with soluble misfolded protein inclusions (Fig. 5F). Our data thus show that Sti1 foci formed during cellular stress do not completely overlap with stress granules or P bodies, but to some extent with Hsp104‐containing protein inclusions (Fig. S7A–C) and extensively with Luc‐GFP inclusions (Fig. 5F).

### Hsp90 aids in clearing Sti1 foci, but is not required for their formation

We sought to determine if the formation of Sti1 foci depends on its Hsp90 function. To this end, cells were treated with Hsp90 ATP hydrolysis inhibitor radicicol at concentrations below the threshold to induce the heat shock response [66]. As reported before [26, 27], we find that the deletion of STI1 significantly reduced growth in cells treated with radicicol, whereas deletion of AHA1 does not cause growth defects. Conversely, overexpression of Sti1 with a low copy number plasmid increased growth in cells treated with radicicol, while overexpression of Aha1 with a low copy number plasmid caused a significant growth reduction in cells treated with radicicol (Fig. 5H). Fluorescence microscopy of yeast cells expressing Sti1‐GFP or Aha1‐GFP grown at 30 °C and heat shocked at 42 °C showed that Aha1‐GFP fluorescence remains diffuse upon radicicol treatment. We also found that radicicol treatment does not prevent the formation of Sti1 foci during heat shock (Fig. 5A). However, when Sti1‐GFP expressing cells recovering at 30 °C from heat shock are treated with radicicol, attenuation of Sti1‐GFP nuclear localization was slowed and attenuation of cytoplasmic foci is significantly reduced (Fig. 5C,D). These results reveal that Sti1 foci formation during heat stress is not disrupted by the inhibition of ATP hydrolysis by Hsp90, whereas inhibition of Hsp90 ATPase function impairs dissolving Sti1 foci during recovery.

### Foci formation is facilitated by the TPR domains of Sti1

We also determined which Sti1 domains are required and sufficient for the formation of cytoplasmic foci upon stress treatment. We expressed C‐terminal GFP fusion proteins of Sti1 TPR2a (amino acid position 222–388), TPR2b (amino acid position 388–519), and TPR2a‐TPR2b (amino acid position 222–519) and monitored the formation of the cytoplasmic foci. High copy number expression of TPR2a‐GFP causes intense fluorescence localized to the nucleus (Fig. 6A,B), which aligns with previous reports that the TPR2a domain of murine STI1 contains a nuclear localization signal [57]. Conversely, high copy number expression of TPR2b‐GFP forms foci that are largely localized to the vacuoles of the yeast. Finally, high copy number expression of TPR2a‐TPR2b‐GFP localizes to both the nucleus and vacuoles and produces similar cytoplasmic foci as observed with HCN expression of full‐length wild‐type Sti1 (Fig. 6A,B), suggesting that foci formation is driven by the combination of the TPR2a and TPR2b domains of Sti1, which can interact with client proteins [67].

### Stip1/hop forms cytoplasmic foci during stress in mammalian cells

**Fig. 7:**
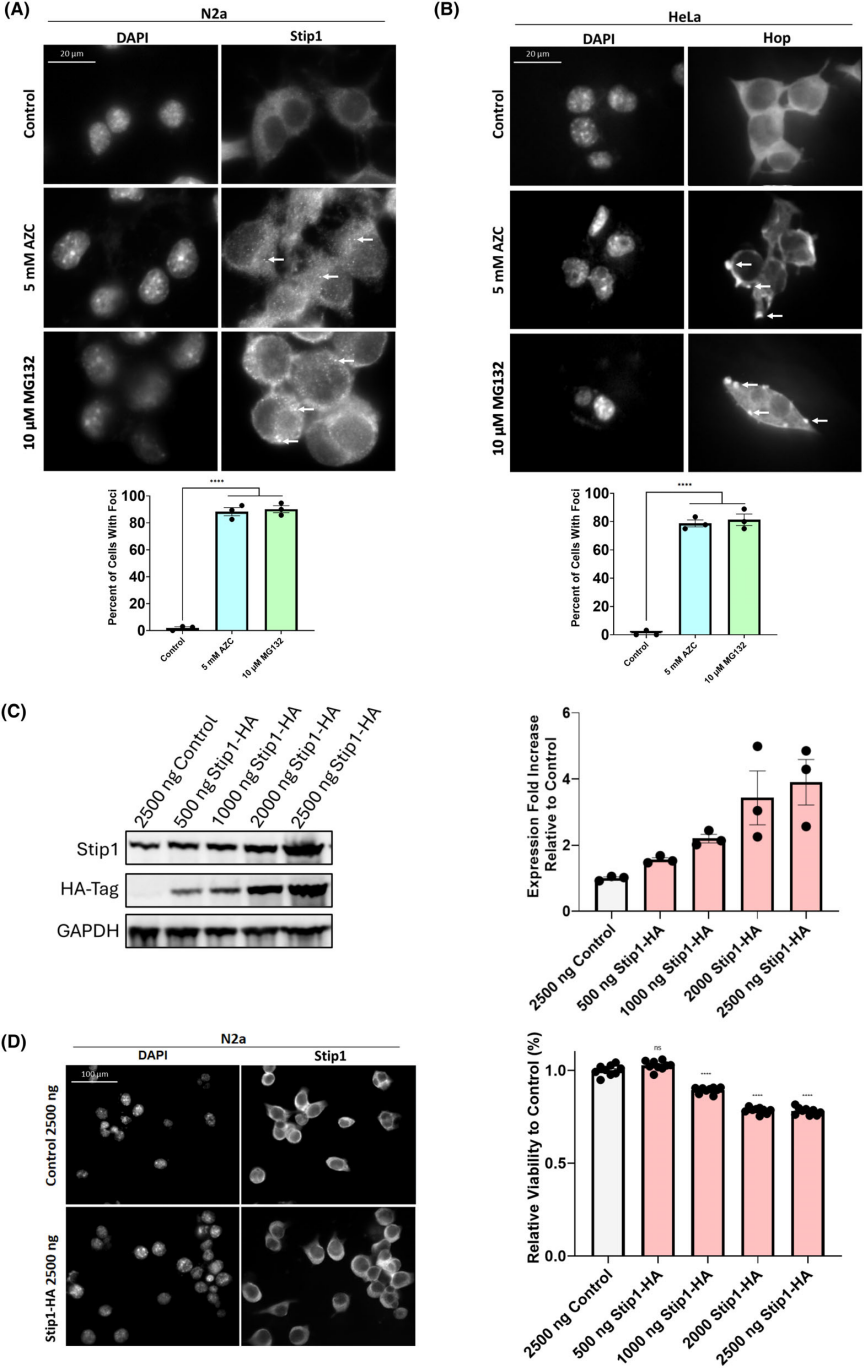
Sti1 changes localization in mammalian cells. (A, B) Immunofluorescence microscopy detecting Stip1 in (A) N2a and (B) Hop in HeLa cells grown under normal growth conditions or treated with either 5 mm L‐azetidine‐2‐carboxylic acid (AZC) or 10 μm MG132 and quantification (below respective images) of Sti1 foci formation (indicated by white arrow) in three independent biological replicates. Scale bars represent 20 μm. (C) Quantification of Stip1 protein levels determined by western blot analysis using anti‐Stip1 and anti‐HA‐Tag primary antibodies in N2A cell lysates from cells transfected with Stip1‐HA expression plasmids at concentrations ranging from 500 to 2500 ng or 2500 ng of a pcDNA 3.1 Empty destination vector and grown under normal growth conditions. Western blots were also probed using an anti‐GAPDH primary antibody as a loading control. Stip1 protein levels was quantified from the anti‐Stip1 blots of cell lysates from three biological replicates. (D) Immunofluorescence microscopy detecting Stip1 (left) and cell viability assay of N2a cells (right) transfected with 2500 ng of a GFP expressing control plasmid or Stip1‐HA expression plasmids at concentrations ranging from 500 to 2500 ng. Cell viability measurements utilized nine biological replicates for each plasmid concentration. To determine statistical significance, unpaired *t*‐tests were used to compare means and standard deviations between relevant controls and experimental data sets (each data set was composed of a minimum of three biological replicates). Statistical significance is represented by an asterisk, where **** is *P* < 0.0001. Error bars represent standard errors of the mean.

We furthermore asked if Stip1/Hop forms similar cytoplasmic foci in mammalian cells as we observed in yeast under stress. Two mammalian cell lines, the mouse N2a and human HeLa cell lines, were cultured under optimal growth conditions and in the presence of AZC or the proteasome inhibitor MG132, and the localization patterns of Stip1, the mouse homolog of Sti1, and Hop, the human homolog of Sti1, were observed using immunofluorescence microscopy. Stip1/Hop localization was diffuse throughout the cytoplasm of both mammalian cell lines in the absence of stress. By contrast, when N2a and HeLa cells were treated with AZC or MG132, distinct Stip1/Hop foci were observed in both cell lines. Previous reports using nuclear export inhibitors suggest Hop nuclear localization is a major component in the Hop stress‐related functions [56]. However, in the absence of export inhibitors, and in contrast to our findings in yeast, we observed no significant nuclear localization upon stress treatment in both cell lines (Fig. 7A,B).

### Proper Stip1 levels are required for mammalian cell viability

Upregulation of HOP has been identified in a variety of human‐derived cancer tissues and cell lines, increasing tumor metastasis and proliferation [39, 68, 69, 70, 71, 72, 73]. Notably, exogenous treatment of Hop to the cell media of ovarian cancer increases markers of cell proliferation [71]. To test if misbalanced Stip1 protein expression affects mammalian cell viability, we transfected N2a cells with increasing amounts of Stip1‐HA expression plasmids. Notably, the Hsp90 and Hsp70 chaperone system is significantly more abundant in HeLa cells than other cell types [74] and as such was not used to assess the overexpression of HOP. We performed immunofluorescence to determine the localization of Stip1 after transfection and cell viability was measured using the ATP levels. The overexpression of Stip1 at the highest transfection levels tested in N2a cells did not cause the formation of stress‐like Sti1 foci as observed in yeast. However, in agreement with the yeast model, we observed a 10% reduction in cell viability upon a 2.2‐fold increase in Stip1 protein levels and cell viability reduced by as much as 20% with a 3.9‐fold increase in Stip1 protein levels (Fig. 7C,D).

## Discussion

**Fig. 8:**
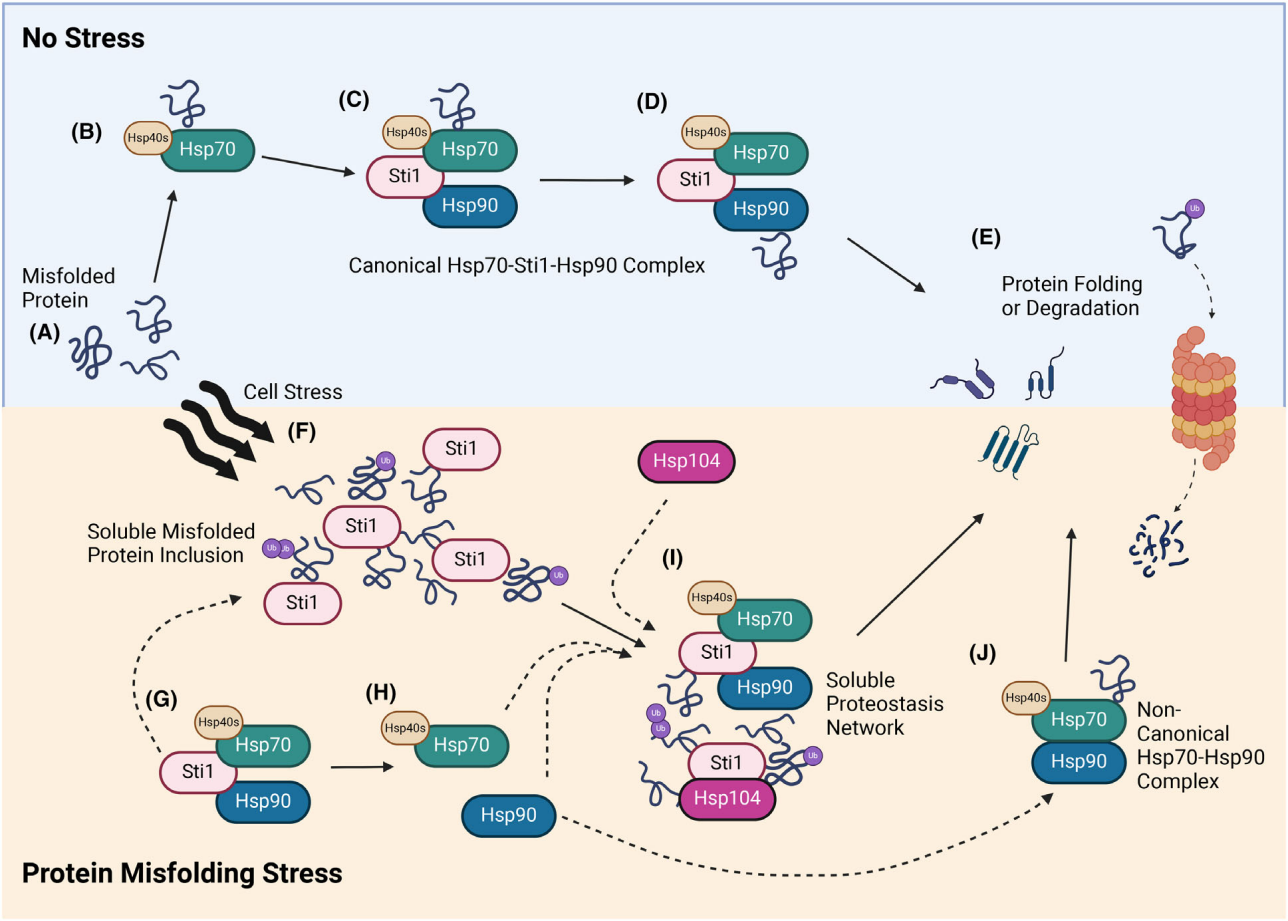
Model of Sti1 recruitment to soluble inclusions comprised of misfolded proteins and other chaperones during stress. (A–C) During periods of low stress, (A) misfolded proteins are recognized by (B) Hsp70 and brought to the (C) Hsp70‐Hsp9‐Sti1 complex. (D, E) Misfolded clients are transferred to Hsp90 to either be (D) refolded or (E) directed to the proteosome for degradation. (F) During periods of high protein misfolding stress, soluble ubiquitinated misfolded proteins accumulate. (G, H) Sti1 is recruited away from the (G) canonical Hsp70‐Hsp9‐Sti1 complex through promiscuous interactions with misfolded proteins and organizes them into areas of high local concentration, leaving a larger population of (H) free Hsp70 and Hsp90. (I) Sti1 then scaffolds the recruitment of other chaperones such as Hsp90, Hsp70, and Hsp104 into soluble Sti1‐mediated proteostasis networks (I) which are effectively cleared by refolding or signaling the misfolded proteins for degradation. (J) Misfolded proteins can also be refolded more efficiently by the noncanonical Hsp70‐Hsp90 complex. Created with BioRender.com.

Sti1 regulates ATP hydrolysis and client transfer within the Hsp90‐Hsp70‐Sti1 complex [75, 76, 77] (Fig. 8A–E). Other functions of Sti1 within proteostasis, however, have remained mostly enigmatic. Here we compare Sti1, which is known to directly interact with client proteins, to Aha1 whose functions are restricted to accelerating Hsp90 ATPase activity [41]. This comparison allowed us to identify the role of Sti1 in proteostasis as a scaffolding protein that recognizes and sequesters misfolded proteins following stress and recruits the proteostasis network to facilitate their clearance.

We find that overexpression of Sti1 reduces the growth of yeast cells and impairs the viability of mammalian cells. Deletion of STI1 and only to a much lesser extent AHA1 causes sensitivity to proteostatic stress and chronological aging. The toxicity of overexpressed Sti1 is exacerbated by the deletion of AHA1 and reduced Hsp90 levels, indicating the precise regulation of Sti1 function within proteostasis, particularly under stress. Notably, upregulation of Sti1 promotes proliferation in multiple cancer cell lines where other members of the proteostasis system are similarly upregulated [39, 68, 69, 70, 71, 72, 73], suggesting that the toxicity associated with exogenous overexpression may stem from increased Sti1 levels independent of an upregulated proteostasis network. Overexpression and ablation of Sti1 and, to a lesser extent Aha1, induces the heat shock response, plausibly due to the accumulation of soluble misfolded proteins and the ensuing sequestration of Hsp70, thus activating Hsf1. Together, our data indicate a central role for strictly controlled levels of Sti1 in proteostasis and induction of the heat shock response, particularly when cells experience proteostatic stress or when the Hsp90 chaperone system is impaired.

Our data also show that Sti1, but not Aha1, drastically changes its subcellular localization upon exposure to proteostatic stress. Rapidly following heat stress, Sti1 shows increased nuclear localization in yeast and the formation of cytoplasmic foci in both yeast and mammalian cells. Sti1 foci do not overlap with known markers of stress granules or P bodies, but partially colocalize with Hsp104, suggesting that Sti1 binds misfolded proteins in the cytoplasm during heat stress and cooperates with Hsp104 in their refolding [14]. Sti1 foci formation is mostly driven by its TPR2 domains most likely via their interactions with misfolded proteins. We also found that stress‐induced Sti1 foci are transient and dissolve rapidly once the stress subsides. Inhibition of Hsp90 ATPase activity significantly prolonged the stability of the Sti1 foci during recovery from stress, indicating that Hsp90, and likely other chaperones are required to refold the misfolded proteins partitioned by Sti1 or triage them for degradation [16]. While previously observed for other chaperones [78, 79, 80], stress‐induced formation of cytoplasmic foci has not been observed for Hsp90 or any of its co‐chaperones with the exception of Sgt2. The interactome of Sti1 changes during periods of high protein misfolding stress to favor interactions with client proteins over its canonical Hsp70 and Hsp90 interactions (Fig. 8G), mediated by its TPR domains [30].

Based on these results, we propose that in acute protein misfolding stress, Sti1 sequesters misfolded proteins through a scaffolding mechanism thereby containing them until they can be refolded by other molecular chaperones or degraded by the ubiquitin proteasome system [8, 81, 82]. The interaction of these misfolded proteins by Sti1 appears to be the first‐rate limiting step, as the formation of cytoplasmic foci of other members of the Hsp70/90/104 and proteasome‐dependent heat‐induced inclusions are dependent on the presence of Sti1 [28]. Interestingly, Stip1/Hop is found in amyloid beta aggregates in mice and humans, and increased levels of Stip1 seem to accelerate amyloidosis in AD mouse models [83]. Similarly, increased levels of Stip1 increase alpha‐synuclein inclusions, and Stip1 binds and colocalizes with alpha‐synuclein in inclusions [84]. These results together indicate that the mechanisms and molecular determinants we uncovered here in yeast seems also to regulate protein misfolding in mammalian cells *in vivo*.

Whether the foci we uncovered represent accumulation of other molecular chaperones is still unknown, but Sti1 can possibly initiate or coordinate the formation of an epichaperome‐like proteostasis network comprised Hsp90, Hsp70 and possibly other molecular chaperones [18, 85] (Fig. 8I). This Sti1‐mediated proteostasis network may also include active proteasomes [21], as we observe an accumulation of soluble ubiquitinated proteins in the absence of Sti1, when the Sti1‐mediated proteostasis network may not form, that are rapidly degraded in wild‐type cells. Alternatively, recruitment of Sti1 into soluble protein inclusions and away from Hsp70 and Hsp90 may initiate the formation of a binary Hsp70‐Hsp90 complex, which can efficiently fold proteins [16, 86] (Fig. 8J).

The formation of epichaperomes has been largely attributed to type one cancer cells where they support survival by chaperoning the unusually high load of newly synthesized proteins [18]. We hypothesize that Sti1 coordinates the formation of a proteostasis network as part of the normal cellular response to protein misfolding stress. However, maladaptive formation of such proteostasis networks may drive aberrant protein–protein interaction in neurodegenerative diseases through a mechanism termed protein connectivity‐based dysfunction [87]. In neurodegenerative diseases, Sti1 as a central protein of such proteostasis networks, may abnormally stabilize misfolded proteins, such as Amyloid‐β [83], α‐synuclein [84], TDP‐43 [88], and PrP^C^ [20], and thus either decrease or increase their toxicity [20, 83, 84] by mechanisms that warrant further investigation. Overall, our work suggests a crucial scaffolding function of Sti1 that contributes to the complex regulation of proteostasis under acute stress and possibly in diseases associated with protein misfolding.

## Materials and methods

### Materials

#### Chemicals

Radicicol and AZC were purchased from Sigma‐Aldrich, St. Louis, MO, USA. Bafilomycin B1 was purchased from Cayman Chemical, Anne Arbor, MI, USA.

#### Yeast strains

Yeast strains BY 4741 (MAT α his3Δ1 leu2Δ0 lys2Δ0 ura3Δ0) and W303 (MAT a leu2‐3112 trp1‐1 can1‐100 ura3‐1 ade2‐1 his3‐11,15 [phi+]) were used in this study. Yeast BY deletion strains were obtained from the Saccharomyces Genome Deletion Project [89], and W303 deletion strains were produced for this study. Yeast strains expressing C‐terminal GFP/mCherry fusion proteins (Sti1‐GFP, Aha1‐GFP, Pab1‐GFP, Edc3‐DsRed) under their endogenous promoters were obtained from the Schuldiner laboratory SWAT [55]. BY‐4741 MAT α cells, expressing Hsf1 mutants under the endogenous Hsf1 promoter were a kind gift from David Pinchus.

#### Yeast media

Yeast–peptone–dextrose (YPD)‐rich media (10 g·L^−1^ of yeast extract, 20 g·L^−1^ of peptone, and 20 g·L^−1^ of dextrose) and selective dextrose (SD) media [6.7 g·L^−1^ yeast nitrogen base (YNB), 60 m·L^−1^ of l‐isoleucine, 20 m·L^−1^ of L‐arginine, 40 m·L^−1^ of L‐lysine HCl, 60 m·L^−1^ of L‐phenylalanine, 10 m·L^−1^ of L‐threonine, and 2% glucose] in either liquid media or agar plates (20 g·L^−1^) were used to grow yeast cells. To produce YPD‐nourseothricin (NAT) plates, 1 L of YPD with agar was supplemented with 100 mg of dissolved nourseothricin before pouring the plates. SD media was supplemented with four different amino acids (40 m·L^−1^ of L‐tryptophan, 60 m·L^−1^ of L‐leucine, 20 m·L^−1^ of L‐histidine‐monohydrate, 10 m·L^−1^ of L‐methionine, and 20 m·L^−1^ of uracil) depending on the selective marker of the plasmid. 2% glycerol or 2% potassium acetate was used instead of glucose as a carbon source for selective glycerol (Sglyc) and selective potassium acetate (SKoAc) media and plates. 2% galactose was used in place of glucose as a carbon source to make selective galactose (SGal) media and plates to induce gene expression from plasmids containing the GAL1 promoter. To expose cells with the established Hsp90 inhibitor, radicicol, liquid media, or plates were supplemented with 10 μg·mL^−1^ of radicicol. To expose cells to the proline analog L‐azetidine‐2‐carboxylic acid (AZC), liquid media or plates were supplemented with 5 mm of AZC.

#### Mammalian cell and media

N2a (RRID:CVCL_0470) mouse neural crest‐derived, HeLa (RRID:CVCL_0030) human adenocarcinoma, and SH‐SY5Y (RRID:CVCL_0019) human neuroblastoma cell lines were used in this study. All cell lines were obtained from the American Type Culture Collection (Manassas, VA, USA). Cells were grown in Dulbecco's modified Eagle medium (DMEM, Corning, Corning, NY, USA) with 4.5 g·L^−1^ glucose. Media was supplemented with 10% fetal bovine serum (FBS, Gibco/Thermo Fisher Scientific, Waltham, MA, USA), 1X penicillin–streptomycin solution (Corning). Cells were grown at 37 °C with 5% CO_2_. The cell lines were authenticated based on cell morphology, cell size, and indicative features. All experiments were performed with mycoplasma‐free cells.

### Methods

#### Deletion of STI1 and AHA1

To produce STI1 and AHA1 deletion W303 yeast strains, homologous recombination was performed using a PCR‐based strategy adapted from Davidson *et al*. [90] to integrate a nourseothricin‐resistance gene in place of the gene (NAT) of interest. The following primers were used: 5′AGTCTGCTCCCAAATTCCTCACTGTAGCTACTAAAACAACCTATACGCAAGAAAGCGTACGCTGCAGGTCGA 3′, Reverse: 5′ TGAAAAAGCAGTAAAAAAAGAATTCAAGATAATAAAGTTATATTTCGTATTATTTATCGATGAATTCGAGCTCG 3′ or Aha1 (Forward: 5′ CGTTATTCCTTTCAGTCTTATTCTTAATCGTTTATAGTAGCAACAATATATCAATCGTACGCTGCAGGTCGA 3′, Reverse: 5′ TAGTGGTATGTAAATATTTACGCATACTTTTATTGAAACATGAGAACAATATATCATCGATGAATTCGAGCTCG 3′). PCR products were transformed into W303 yeast cells and allowed to recover for 4 h in YPD at 30 °C before being plated on YPD‐NAT plates. Colonies were selected, and the genomic DNA was extracted using lithium acetate‐SDS lysis and DNA purification with ethanol precipitation as described by Looke *et al*. [91] to confirm proper gene deletion. PCR amplification primers: Forward: 5′ TCTTCGCTTCTCCTCCTTTAAGGAATAAAG 3′ Reverse: 5′ GTTCCGATTTCTCAAATATCAACCATAGCA 3′ and Aha1 (Forward: 5′ CACTCGTATAGGAAACTTTGATAGAGAGGC 3′ Reverse: 5′ GCCGTACAGAACTACATGATGTATTGCATT 3′).

#### Yeast transformations

Yeast transformations were performed utilizing the standard PEG/lithium acetate transformation method [92].

#### Growth assays (spotting assays) and quantification

The relative growth of the yeast cells was measured using spotting assays and quantification as described by Petropavlovskiy *et al*. [93]. In short, cells were inoculated into 3 mL of SD media and incubated in a shaking incubator at 30 °C overnight. Normalized serial dilutions of cells were spotted on to SD, SGlyc, SKOAc, or SGal plates lacking the selective amino acid markers using a 48‐prong frogger (V&P Scientific, San Diego, CA, USA). For AZC and radicicol‐treated plates, AZC or radicicol was added to SD media to a final concentration of 5 mm or 10 μg·mL^−1^, respectively, immediately before media was poured when warm to the touch. Plates were incubated at either 30 or 37 °C, and the growth of the yeast colonies was monitored during the entire growth period All spotting assays contain respective control lanes on each experimental plate, and images of the plates were cropped and separated to highlight relative side‐by‐side comparisons. Unless otherwise indicated, the pixel count of the third dilution of the growth assay was used for quantification. Values were normalized to the average of the respective control lanes. For all growth assays, a minimum of three biological replicates were used.

#### Fluorescent microscopy and quantification

Microscopy imaging of fluorescent‐tagged constructs was performed by first inoculating cells in SD media and incubating overnight at 30 °C in a shaking incubator. To induce heat shock, cultures were incubated at 42 °C for 1 h unless otherwise indicated. For constructs under transcriptional control of the GAL1 promoter, overnight cultures were washed twice with sterile water and then grown in SGal media for 6 h prior to performing microscopy. Small volumes (1–2 μL) of each culture were placed on a microscope slide and imaged using a Zeiss Axio Vert.A1 microscope. For each condition, three biological replicates were imaged, and three images of random fields of each biological replicate were imaged. The extent of foci formation was quantified as the percentage of cells that contained foci and the extent of nuclear localization was categorized by the percent of cells displaying significant fluorescence in the nucleus [94, 95, 96]. To measure the attenuation of foci and nuclear localization, cells were incubated in the absence or presence of 10 μg·mL^−1^ radicicol at 30 °C after a 1‐h 42 °C heat shock and samples were collected at subsequent time points. Each data point represents the average percentage of cells that contained foci or nuclear localization across the three images taken for each respective biological replicate. For each biological replicate, a minimum of 100 cells was counted toward the percent of cells containing foci unless otherwise stated.

#### Stress reporter assays

Cells were transformed plasmids containing GFP downstream of eight consecutive stress response element enhancers (STRE) adjacent to a crippled Cyc1 promoter driving expression of GFP or four heat shock response element enhancers (HSRE) with identical features [46]. Single colonies were used to inoculate 3 mL of SD media and grown overnight at 30 °C in a shaking incubator. Cells were washed twice with sterile water, and 50 μL of each cell culture was diluted with 150 μL of sterile 1× phosphate buffer saline (Wisent Inc, Saint‐Jean‐Baptiste, QC, Canada) in a 96‐well plate. The initial OD600 and fluorescence (Excitation: 500/20, Emission: 541/20) of each well were measured using a Cytation 5 Cell Imaging Multi‐Mode Reader (Biotek, Winooski, VT, USA). The plate was then incubated at 42 °C with continuous double orbital shaking at 600 rpm for 3 h, and the fluorescence of each well was measured at 10‐min intervals. For all conditions, a minimum of three biological replicates were used and each biological replicate represents an average of four technical repeats.

#### Alkaline protein lysis of yeast cells

Single yeast colonies were used to inoculate 3 mL of SD media and grown overnight at 30 °C in a shaking incubator. To produce heat‐shocked lysates, cultures were incubated at 42 °C in a shaking incubator for 1 h before performing lysis. Cultures were centrifuged for 4 min at 3000 ***g***, and the supernatant was aspirated. Pellets were resuspended in 200 μL of alkaline lysis buffer (0.1 m NaOH, 0.05 m EDTA, 2% SDS, and 2% BME), and the OD600 was recorded for the purpose of normalization. The resuspension was incubated for 10 min at 90 °C, and 67 μL of 4× Laemmli sample buffer (90% Laemmli sample buffer and 10% BME) was added. Samples were centrifuged to pellet cellular debris before loading on SDS/PAGE gel.

#### Western blotting

SDS/PAGE was performed with the samples using 8–16% gradient gels Bio‐Rad Criterion TGX Stain‐Free Precast Gels (Bio‐Rad, Hercules, CA, USA) run at 200 V for about 30 min. Proteins were transferred onto nitrocellulose membranes (Bio‐Rad) using the Bio‐Rad Trans‐Blot Turbo machine following the manufacturer's protocol. Membranes were blocked using 5% skim milk powder (Carnation/Nestle, Vevey, Switzerland) in Tris‐buffered saline with 0.05% (v/v) Tween‐20 (Thermo Fisher Scientific) (TBST) and incubated for 2 h on a shaker at room temperature. Following blocking, the membrane was washed with 10 mL aliquots of TBST for 10‐min intervals over a 30‐min period. The membrane was then incubated overnight with primary antibody diluted in TBST with 1% (w/v) Bovine serum albumin (BSA) (Wisent Inc) on a shaking incubator at 4 °C. The membrane was washed again using 10 mL aliquots of TBST at 10‐min intervals for 30 min and then incubated in secondary antibody diluted in TBST with 1% (w/v) bovine serum albumin on a shaker for 1 h at room temperature. Following incubation, the membrane was washed one final time in 10 mL of TBST and the membrane was imaged using the ChemiDoc MP Imaging System (Bio‐Rad) and analyzed using Image Lab (Bio‐Rad).

#### Sedimentation assay

Sedimentation assay protocol was adapted from Theodoraki *et al*. and Shiber *et al*. [97, 98]. Five milliliters of YPD media was inoculated with one yeast colony and grown overnight at 30 °C. The culture was then centrifuged at 3000 ***g***, washed with water, and then pelleted and resuspended in 200 μL lysis buffer [100 mm Tris pH 7.5, 200 mm NaCl, 5% glycerol, 5 mm EDTA, 1 mm DTT, 10 mm Phenanthroline, 1x ProBlock Gold Yeast Protease Inhibitor Cocktail (GoldBio, St. Louis, MO, USA)] and transferred into an Eppendorf tube. Acid‐washed glass beads (425–600 μm, Sigma) were added, and cells were physically lysed using a vortex mixer for six 30‐s intervals and cooled on ice for 30 s between each interval. A 16‐gauge needle was used to pierce the tubes, and the lysates were collected in a clean Eppendorf tube by centrifugation. A BCA protein assay was performed according to the Thermo Scientific Pierce BCA Protein Assay Kit (Thermo Fisher Scientific) to determine the total protein concentration of the lysates. The total lysate was centrifuged at 500 ***g*** for 15 min at 4 °C, and the supernatant was transferred to a fresh tube and combined with an equal volume of SUMEB Buffer (8 m Urea, 1% SDS, 10 mm MOPS, 10 mm EDTA, and 0.01% bromophenol blue). The pellet was resuspended in 150 μL of lysis buffer combined with an equal volume of SUMEB Buffer. The samples were boiled at 95 °C for 5 min, and the BCA assay concentrations were used to load equal concentrations of sample onto 8–16% gradient gels Bio‐Rad Criterion TGX Stain‐Free Precast Gels (Bio‐Rad) for SDS/PAGE, as described above. The gels were imaged using the stain‐free protocol on the ChemiDoc MP Imaging System (Bio‐Rad) and analyzed using Image Lab (Bio‐Rad). Western blotting was performed as described above.

#### Mammalian cell transfection

Mammalian cells were grown to approximately 90% confluency in DMEM + 10% FBS + 1% penstrep and transfected in Opti‐MEM (Gibco/Thermo Fisher Scientific) low serum media with lipofectamine 3000 with p3000 reagent (Thermo Fisher Scientific), following supplier recommended concentrations. Cells were incubated at 37 °C in 5% CO_2_ for 6 h and then washed with 1× PBS and incubated in DMEM + 10% FBS + 1% penstrep for 16 h at 37 °C. Cells were subsequently separated into different dishes for each experimental setup.

#### Mammalian cell viability assay

Cell viability was measured using the CellTiter‐Glo 2.0 Luminescent Cell Viability Assay (Promega, Madison, WI, USA). Cells were split into a 96‐well plate and grown for 24 h at 37 °C in DMEM without treatment (Control cells) or DMEM with 10 mm AZC, 10 μm MG132, or 20 μm radicicol for 18 h depending on the experimental setup. Each well was seeded with 10 000 cells. The CellTiter‐Glo 2.0 Luminescent Cell Viability Assay was carried out following the supplier's instructions and fluorescence was measured using a Cytation 5 Cell Imaging Multi‐Mode Reader (Biotek).

#### Immunofluorescence microscopy

N2a and HeLa cells were split into 12‐well plates containing glass cover slips treated with poly‐L‐lysine and grown in DMEM + 10% FBS + 1% penstrep without treatment (Control Cells) at 37 °C for 48 h or supplemented with 5 mm AZC or 10 μm MG132 during the final 18 h of incubation. Each well was seeded with 30 000 cells. The DMEM + 10% FBS + 1% penstrep media was aspirated off, and cells were washed twice with PBS. 4% paraformaldehyde in PBS was added to each well and cells were incubated for 15 min at 37 °C for fixation. Cells were washed three times with ice‐cold PBS, letting cells sit in each wash for 3 min. The cells were then incubated for 1 h at room temperature in a solution of 10% normal goat serum (Gibco), 0.1% Triton X‐100, and PBS to block nonspecific binding and permeabilize the cells. The blocking solution was aspirated off and replaced with fresh blocking solution containing the primary antibody and incubated at 4 °C overnight. Cells were washed three times with PBS and incubated for 1 h at room temperature in blocking solution containing secondary antibody. Cells were washed three times with PBS, all liquid was aspirated, and coverslips were mounted with ProLong® Gold Antifade Mountant with DAPI (Invitrogen, Carlsbad, CA, USA). The cells were imaged using a Zeiss Axio Vert.A1 microscope. For quantification of cytoplasmic foci, each data point represents the average percentage of cells that contained foci across the five images taken for each respective biological replicate. For each biological replicate, a minimum of 80 cells was counted toward the percent of cells containing foci unless otherwise stated.

#### Protein expression induction by heat shock and protease inhibition

SH‐SY5Y cells were cultured in DMEM + 10% FBS + 1% penstrep at 37 °C in 5% CO_2_ to approximately 90% confluency. Six well culture plates were seeded with 400 000 cells per well and allowed to attach and grow for 48 h while incubating in DMEM + 10% FBS + 1% penstrep at 37 °C in 5% CO_2_. Cells were then incubated for 24 h in media containing one of the following treatments: 1 μm MG132, 50 nm bafilomycin B1, 1 μm MG132 + 50 nm bafilomycin B1, 500 nm radicicol, or 2.5 mm AZC. Cells were lysed using a RIPA lysis buffer containing: 150 mm NaCl, 1% Triton X‐100, 0.1% SDS, 20 mm Tris pH 7.5, 1× Halt protease inhibitors (Thermo Fisher Scientific), 1× EDTA. A BCA protein assay was performed according to the Thermo Scientific Pierce BCA Protein Assay Kit (Thermo Fisher Scientific) to determine the total protein concentration in the total lysates.

### Statistical analysis

Statistical analysis of growth assays, percentage of foci assessed by microscopy, and western blots were performed using the graphpad prism *(GraphPad Inc, San Diego, CA, USA) software. To determine statistical significance, unpaired *t*‐tests were used to compare means and standard deviations between relevant controls and experimental data sets (each data set was composed of a minimum of three biological repeats). Statistical significance is represented by an asterisk, where **** is *P* < 0.0001, *** is *P* < 0.001, ** is *P* < 0.01, and * is *P* < 0.05. Error bars represent standard errors of the mean.

## Author contributions

BSR, YJK, DWM, and JCJ‐C performed experiments and together with MAMP, JLJ, W‐YC, and MLD, contributed key reagents, data analyses, interpretation, and critical assessment. BSR, W‐YC, and MLD designed the study and wrote the paper. All authors read and approved the final manuscript.

## Conflict of interest

The authors declare no conflict of interest.

## Peer review

The peer review history for this article is available at https://www.webofscience.com/api/gateway/wos/peer‐review/10.1111/febs.17389.

## Supporting information


**Fig. S1.** STI1 and AHA1 deletion does not affect growth on alternative carbon sources.
**Fig. S2**. Deletion of STI1 and AHA1 alter the growth of W303 yeast during aging.
**Fig. S3**. Relative Sti1 protein levels induced by LCN and HCN Sti1 plasmids.
**Fig. S4**. Low overexpression of Sti1 and Aha1 causes no growth phenotypes on alternative carbon sources.
**Fig. S5**. Induction of Hsp70 protein expression in SH‐SY5Y cells.
**Fig. S6**. Deletion of STI1 and AHA1 cause a similar accumulation of misfolded soluble proteins during stress and heat shock does not alter the sedimentation profile of Sti1.
**Fig. S7**. Sti1‐GFP only partially colocalizes with other compartments of misfolded proteins during stress.

## Data Availability

All data are available in the main text or the Supplementary Materials upon reasonable request.
