# Supplementary material for: Stress‐inducible phosphoprotein 1 (Sti1/Stip1/Hop) sequesters misfolded proteins during stress
Source: FEBS J. 2024 Dec 30;292(14):3634–58. doi: 10.1111/febs.17389 (PMC12265868; doi:10.1111/febs.17389)
Supplement: Supplementary file 1 — Fig. S1. STI1 and AHA1 deletion does not affect growth on alternative carbon sources. Fig. S2. Deletion of STI1 and AHA1 alter the growth of W303 yeast during aging. Fig. S3. Relative Sti1 protein levels induced by LCN and HCN Sti1 plasmids. Fig. S4. Low overexpression of Sti1 and Aha1 causes no growth phenotypes on alternative carbon sources. Fig. S5. Induction of Hsp70 protein expression in SH‐SY5Y cells. Fig. S6. Deletion of STI1 and AHA1 cause a similar accumulation of misfolded soluble proteins during stress and heat shock does not alter the sedimentation profile of Sti1. Fig. S7. Sti1‐GFP only partially colocalizes with other compartments of misfolded proteins during stress. [file FEBS-292-3634-s001.pdf]

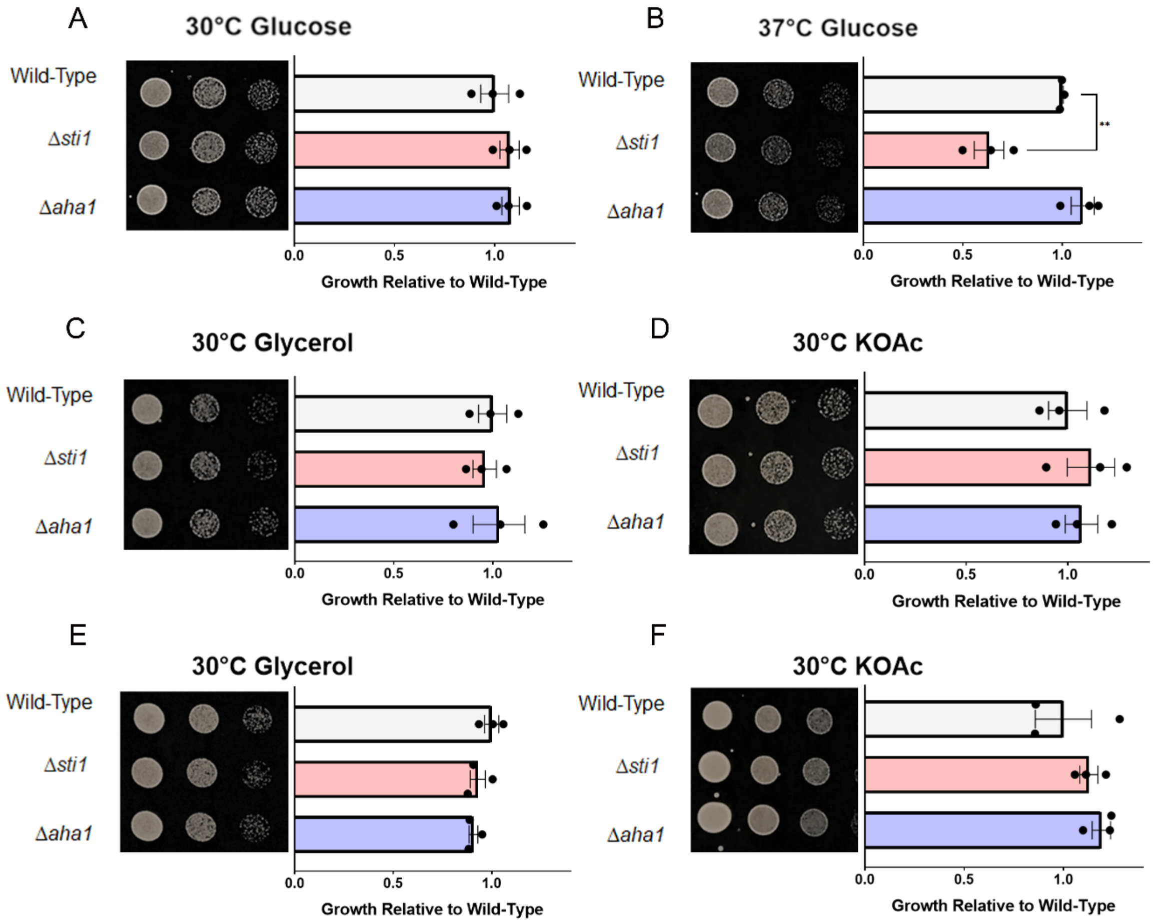

Fig. S1. STI1 and AHA1 deletion does not affect growth on alternative carbon sources. (A-B) Saccharomyces cerevisiae spotting assay of Wild type, Δsti1, and Δaha1 BY 4741 yeast grown at optimal growth temperatures (30 °C) (A) and heat stress conditions (37 °C) (B) on SD media. (C-D) Spotting assays of Wild type, Δsti1, and Δaha1 BY 4741 yeast grown at optimal growth temperatures on SD glycerol (C) and SD KOAc (D). (E-F) Spotting assays of Wild type, Δsti1, and Δaha1 W303 yeast grown at optimal growth temperatures on SD glycerol (E) and SD KOAc (F). Cells are spotted in three five-fold dilutions from left to right and toxicity is inferred by lack of growth. To determine statistical significance, unpaired t-tests were used to compare means and standard deviations between relevant controls and experimental data sets (each data set was composed of a minimum of three biological replicas). Statistical significance is represented by an asterisk, where **** is P < .0001, *** is P < .001, ** is P < .01, and * is P < .05. Error bars represent standard errors of the mean.

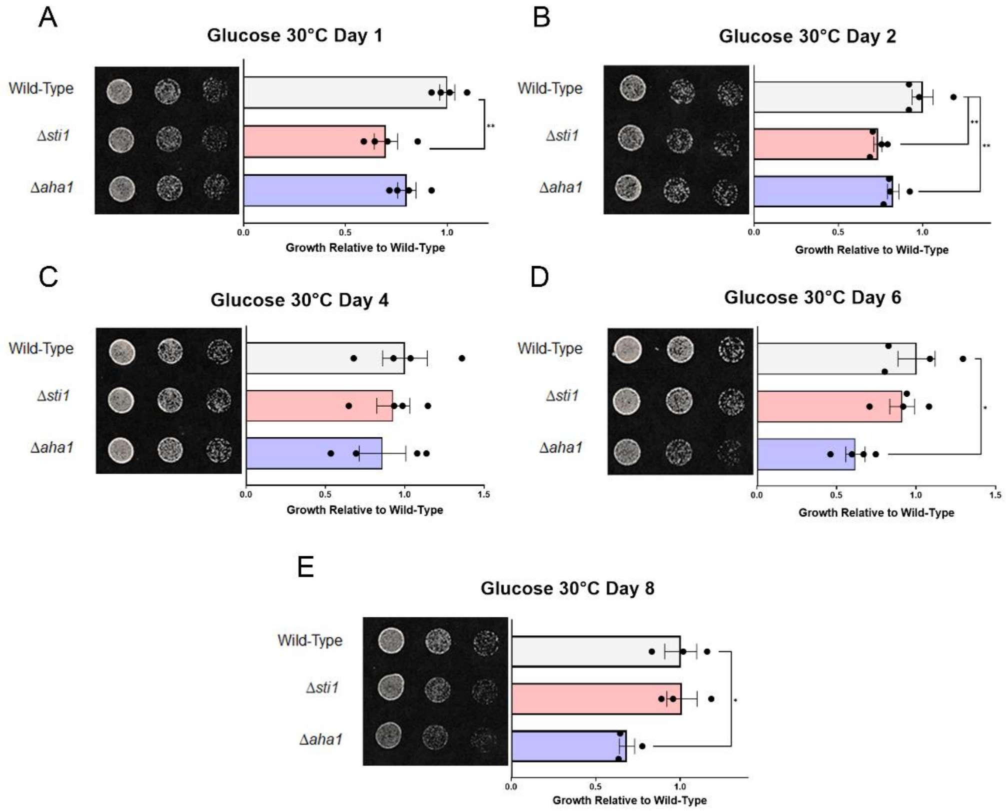

Fig. 2. Deletion of STI1 and AHA1 alter the growth of W303 yeast during aging. (A-E) Saccharomyces cerevisiae spotting assay of Wild type, Δsti1, and ΔAha1 W303 yeast grown at optimal growth temperatures (30 °C) on SD media after incubation in SD media for an additional (A) 1, (B) 2, (C) 4, (D) 6, or (E) 8 days beyond the standard overnight growth. Cells are spotted in three five-fold dilutions from left to right and toxicity is inferred by lack of growth. To determine statistical significance, unpaired t-tests were used to compare means and standard deviations between relevant controls and experimental data sets (each data set was composed of a minimum of three biological replicas). Statistical significance is represented by an asterisk, where **** is P < .0001, *** is P < .001, ** is P < .01, and * is P < .05. Error bars represent standard errors of the mean.

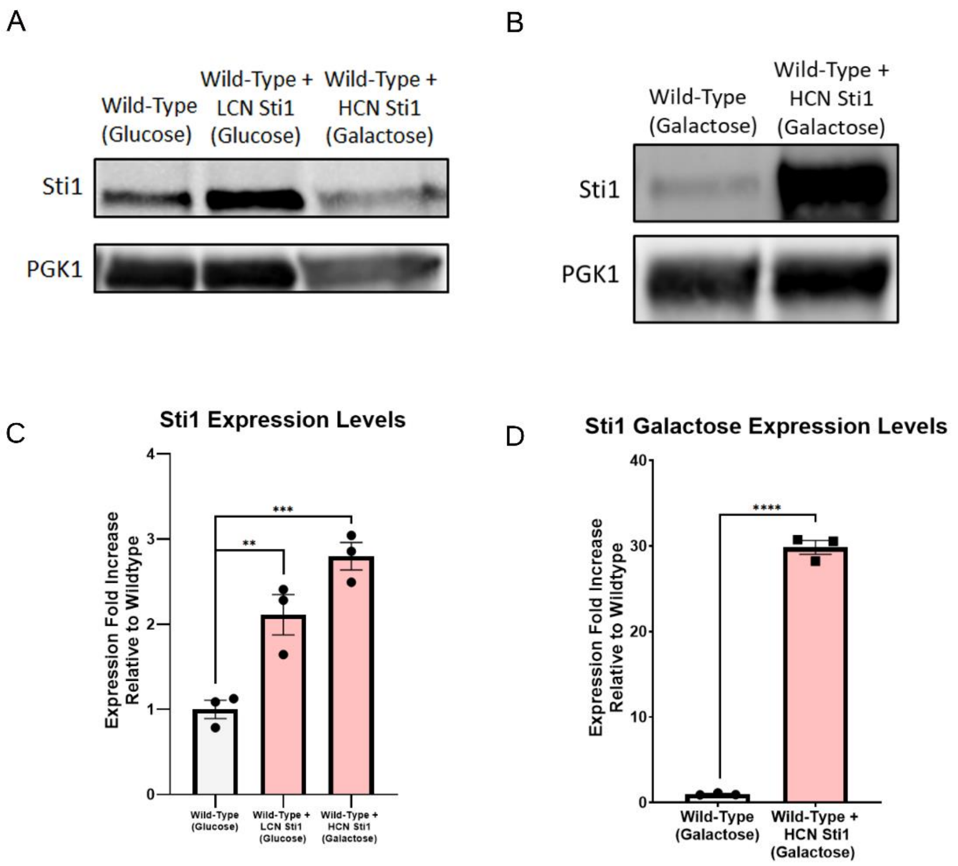

Fig. S3. Relative Sti1 protein levels induced by LCN and HCN Sti1 plasmids. Quantification of Sti1 protein levels induced by transformation of LCN and HCN Sti1 expression systems relative to basal Sti1 protein levels in W303 cells grown in glucose (A) or galactose (B) at optimal growth temperatures (30 °C) by western blot. Western blots were also probed using an anti-PGK1 primary antibody for loading control that was subsequently used to normalize Sti1 protein levels in quantification. Comparison of Sti1 protein levels in W303 transformed with LCN Sti1 and grown in glucose or W303 transformed with HCN Sti1 and grown in galactose relative to basal Sti1 expression in W303 cells grown in glucose (C). Comparison of Sti1 levels in W303 transformed with HCN Sti1 and grown in galactose relative to basal Sti1 expression in W303 cells grown in galactose (D). To determine statistical significance, unpaired t-tests were used to compare means and standard deviations between relevant controls and experimental data sets (each data set was composed of a minimum of three biological replicas). Statistical significance is represented by an asterisk, where **** is $P < .0001$, *** is $P < .001$, ** is $P < .01$, and * is $P < .05$. Error bars represent standard errors of the mean.

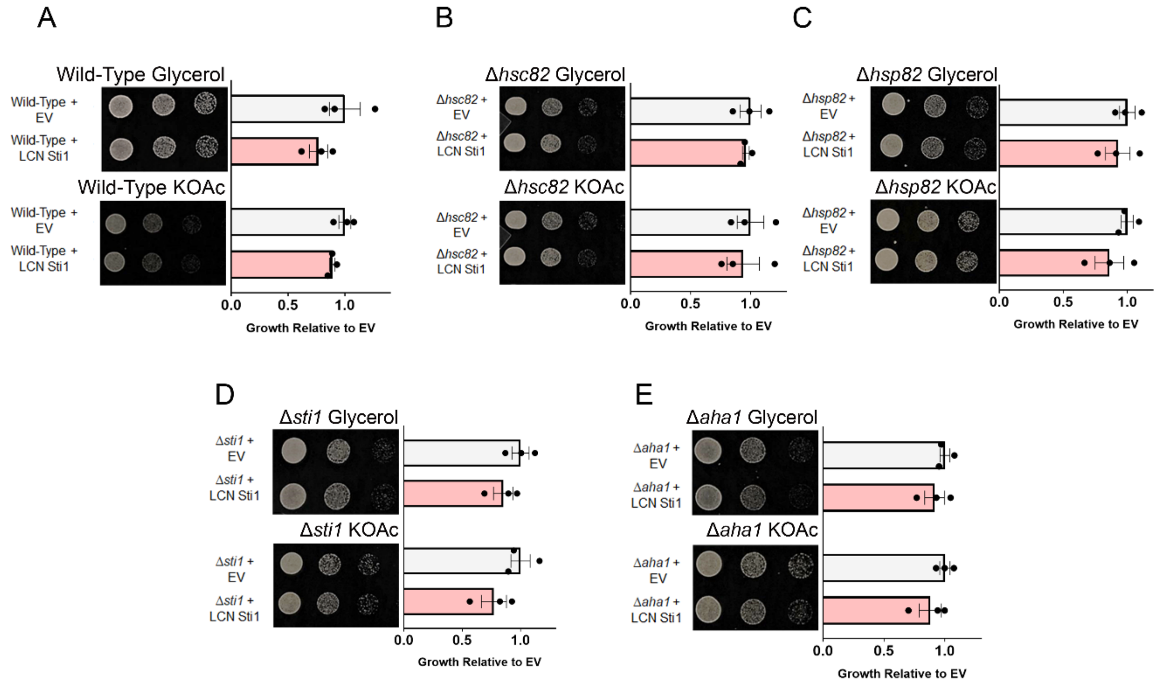

Fig. S4. Low overexpression of Sti1 and Aha1 causes no growth phenotypes on alternative carbon sources. Saccharomyces cerevisiae spotting of Wild type (A), ΔHsp82 (B), ΔHsc82 (C), Δsti1 (D), and Δaha1 (E) BY-4741 yeast transformed with low copy number (LCN) EV or LCN expression plasmids of Sti1 and grown at optimal growth temperatures (30 °C) on Sglyc and SKOAc media. Cells are spotted in three five-fold dilutions from left to right and toxicity is inferred by lack of growth in the Sti1 expressing lines. To determine statistical significance, unpaired t-tests were used to compare means and standard deviations between relevant controls and experimental data sets (each data set was composed of a minimum of three biological replicas). Statistical significance is represented by an asterisk, where **** is P < .0001, *** is P < .001, ** is P < .01, and * is P < .05. Error bars represent standard errors of the mean.

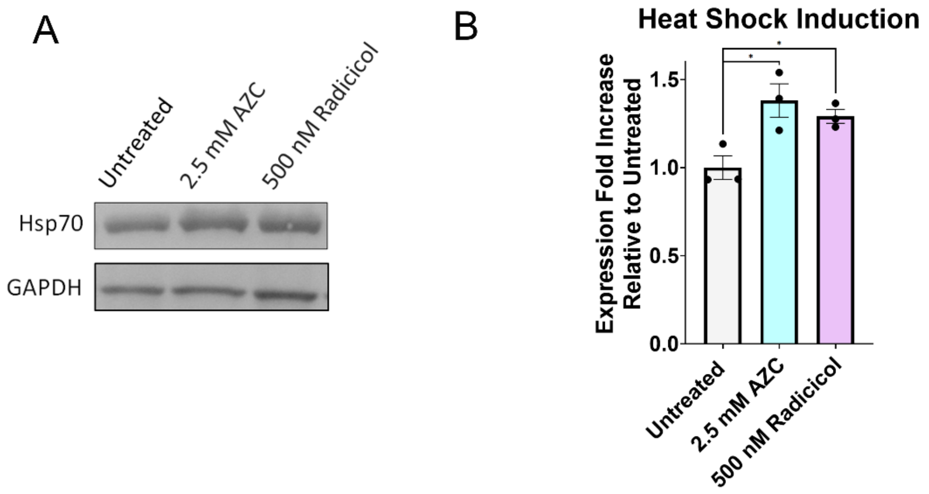

Fig. S5. Induction of Hsp70 protein expression in SH-SY5Y cells. Quantification of Hsp70 protein levels determined by western blot analysis using an anti-Hsp70 primary antibody in SH-SY5Y cell lysates from cells grown under normal growth conditions or treated with reagents to induce heat shock. Western blots were also probed using an anti-GAPDH primary antibody for loading control that was subsequently used to normalize sti1 protein levels in quantification. To determine statistical significance, unpaired t-tests were used to compare means and standard deviations between relevant controls and experimental data sets (each data set was composed of a minimum of three biological replicas). Statistical significance is represented by an asterisk, where **** is $P < .0001$, *** is $P < .001$, ** is $P < .01$, and * is $P < .05$. Error bars represent standard errors of the mean.

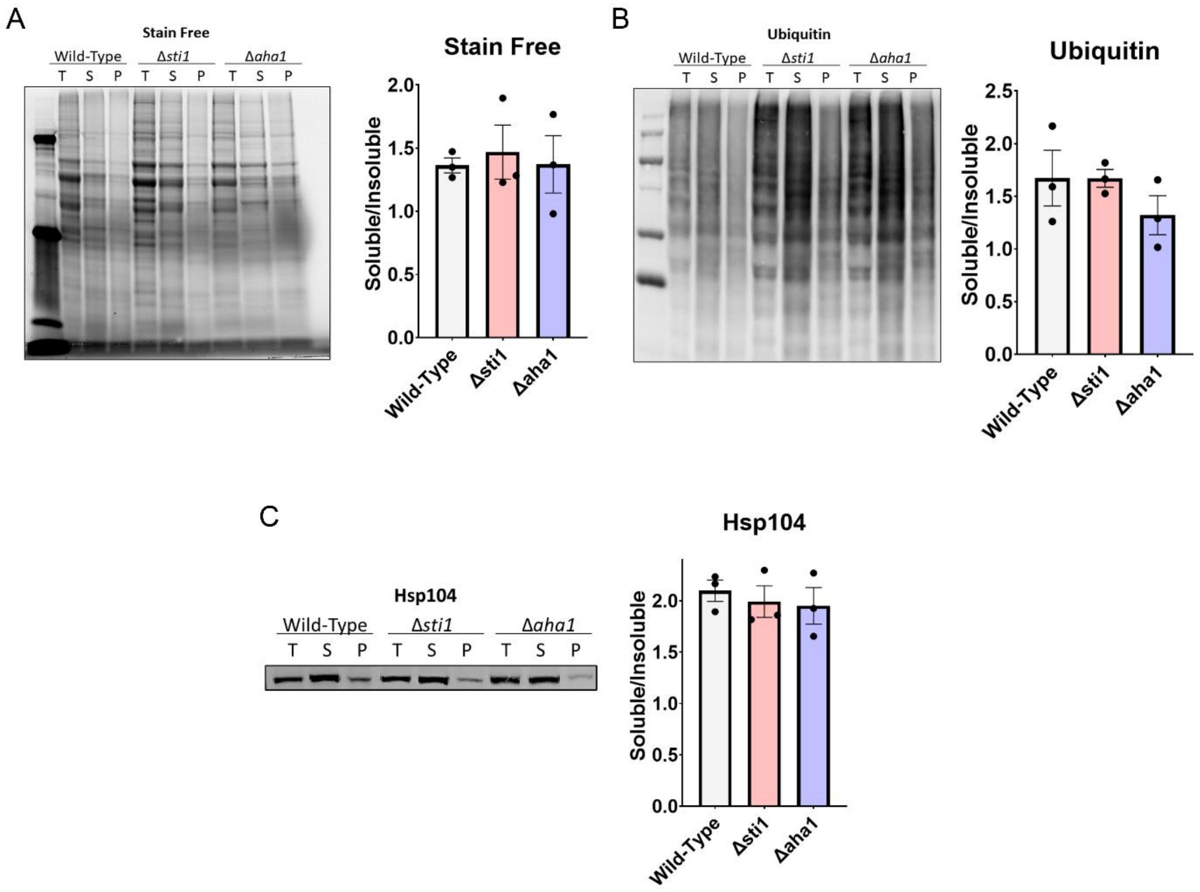

Fig. S6. Deletion of STI1 and AHA1 cause a similar accumulation of misfolded soluble proteins during stress and heat shock does not alter the sedimentation profile of Sti1. (A-C) Sedimentation analysis of cell lysates from Wild type, Δ*sti1*, and Δ*Aha1* yeast (W303) grown in YPD and heat shocked for one hour at 42 °C. Fractionated lysates were imaged with (A) stain free gels and probed with (B) anti-ubiquitin, and (C) anti-Hsp104 primary antibodies for quantification of sedimentation profile. To determine statistical significance, unpaired t-tests were used to compare means and standard deviations between relevant controls and experimental data sets (each data set was composed of a minimum of three biological replicas). Statistical significance is represented by an asterisk, where **** is $P < .0001$, *** is $P < .001$, ** is $P < .01$, and * is $P < .05$. Error bars represent standard errors of the mean.

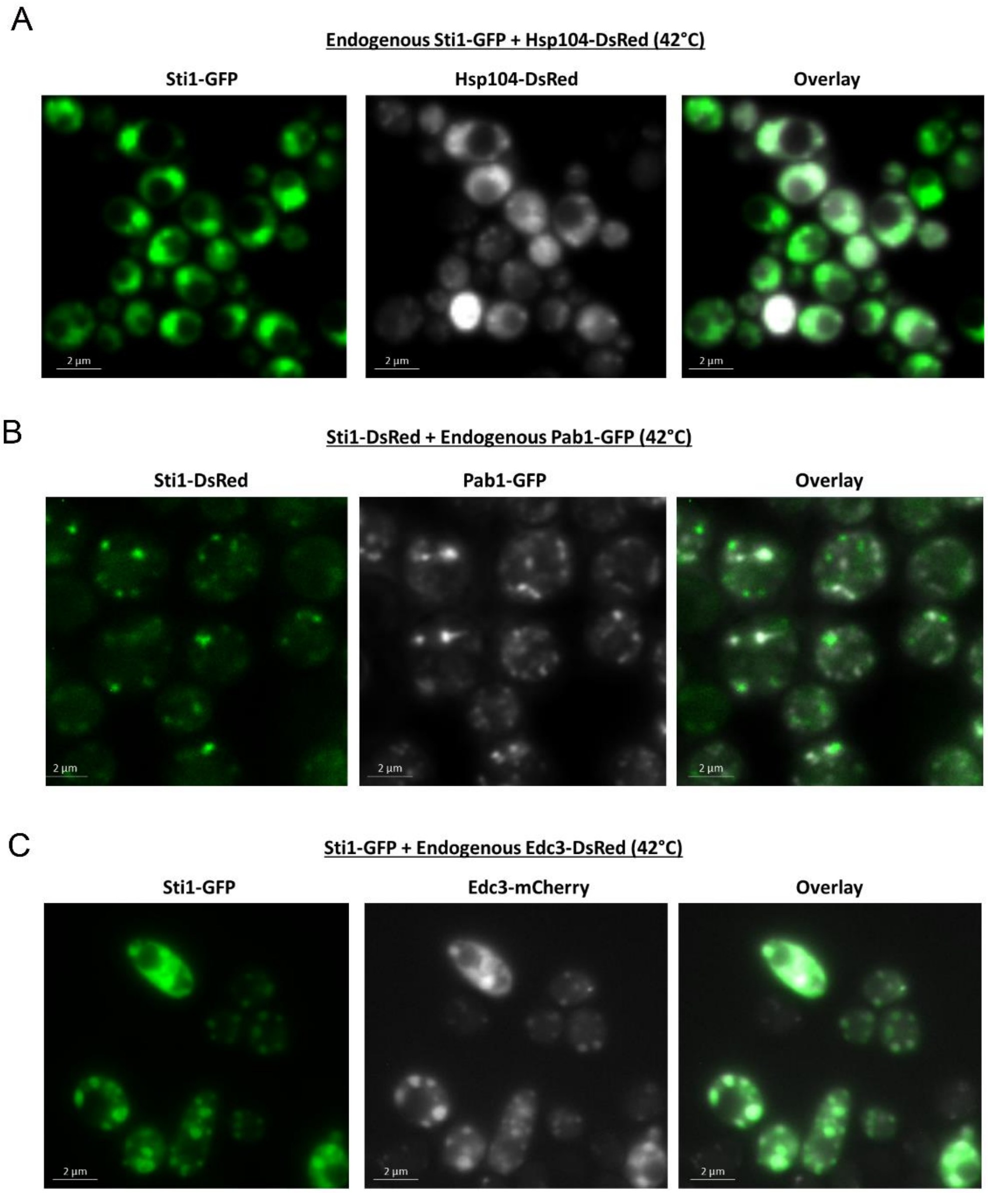

Fig. S7. Sti1-GFP only partially colocalizes with other compartments of misfolded proteins during stress. (A) Fluorescence microscopy after one hour of heat shock (42 °C) of BY 4741 yeast expressing Sti1-GFP under its endogenous promoters and the protein aggregation marker Hsp104-RFP on a transformed plasmid. (B) Fluorescence microscopy after one hour of heat shock (42 °C) of BY 4741 yeast expressing the stress granule marker PAB1-RFP under its endogenous promoter and transformed with LCN Sti1-GFP plasmid. (C) Fluorescence microscopy after one hour of heat shock (42 °C) of BY 4741 yeast expressing the P body marker Edc3-RFP under its endogenous promoter and transformed with LCN Sti1-GFP plasmid. Yeast cells were grown in SD media overnight prior to heat shock.
